# Quantitative capabilities of four state-of-the-art SPECT-CT cameras

**DOI:** 10.1186/2191-219X-2-45

**Published:** 2012-08-27

**Authors:** Alain Seret, Daniel Nguyen, Claire Bernard

**Affiliations:** 1 Imagerie Médicale Expérimentale, Cyclotron Research Centre, Université de Liège, Liège, 4000, Belgium; 2 Department of Physics, Université de Liège, Liège, 4000, Belgium; 3 Nuclear Medicine, University Hospital (CHU Liège), Université de Liège, Liège, 4000, Belgium

**Keywords:** SPECT-CT, Iterative, Resolution, Quantification, Contrast

## Abstract

**Background:**

Four state-of-the-art single-photon emission computed tomography-computed tomography (SPECT-CT) systems, namely Philips Brightview, General Electric Discovery NM/CT 670 and Infinia Hawkeye 4, and Siemens Symbia T6, were investigated in terms of accuracy of attenuation and scatter correction, contrast recovery for small hot and cold structures, and quantitative capabilities when using their dedicated three-dimensional iterative reconstruction with attenuation and scatter corrections and resolution recovery.

**Methods:**

The National Electrical Manufacturers Association (NEMA) NU-2 1994 phantom with cold air, water, and Teflon inserts, and a homemade contrast phantom with hot and cold rods were filled with ^99m^Tc and scanned. The acquisition parameters were chosen to provide adequate linear and angular sampling and high count statistics. The data were reconstructed using Philips Astonish, General Electric Evolution for Bone, or Siemens Flash3D, eight subsets, and a varying number of iterations. A procedure similar to the one used in positron emission tomography (PET) allowed us to obtain the factor to convert counts per pixel into activity per unit volume.

**Results:**

Edge and oscillation artifacts were observed with all phantoms and all systems. At 30 iterations, the residual fraction in the inserts of the NEMA phantom fell below 3.5%. Contrast recovery increased with the number of iterations but became almost saturated at 24 iterations onwards. In the uniform part of the NEMA and contrast phantoms, a quantification error below 10% was achieved.

**Conclusions:**

In objects whose dimensions exceeded the SPECT spatial resolution by several times, quantification seemed to be feasible within 10% error limits. A partial volume effect correction strategy remains necessary for the smallest structures. The reconstruction artifacts nevertheless remain a handicap on the road towards accurate quantification in SPECT and should be the focus of further works in reconstruction tomography.

## Background

In the decade following the development of the first hybrid single-photon emission computed tomography-computed tomography (SPECT-CT) system, the manufacturers have progressively introduced fully integrated SPECT-CT systems to the market [1]. Over approximately the same period, they firstly introduced two-dimensional (2D) iterative reconstructions as a replacement for filtered back-projection (FBP); secondly, they added attenuation and scatter corrections, and finally, in very recent years, they turned to three-dimensional (3D) iterative reconstructions, which include attenuation and scatter corrections and resolution recovery.

Although the commercial SPECT-CT systems (Table 1) and 3D reconstruction algorithms (Table 2) of the three major manufacturers have common features, they also differ in many points. In each case, the SPECT component uses similar technologies still based on the 50-year-old Anger camera. Furthermore, the crystal, the number of photomultiplier tubes, and the planar spatial resolution are strictly identical between the four systems, and the fields of view are approximately identical. However, the CT components are much more different. The General Electric Hawkeye 4 (GE Healthcare, Waukesha, VI, USA) CT is a low-dose four-slice CT with very low tube current, slow rotation (about 20 s), and imaging characteristics that, especially for the spatial resolution, are far from those of a modern state-of-the-art spiral multi-slice CT like the one found in the General Electric Discovery NM/CT 670. The CT components of the Siemens Symbia T2 and T6 (Siemens Medical Solutions USA, Hoffman Estates, IL, USA) are somehow intermediate systems between the Hawkeye and a state-of-the-art CT as they use a high-current CT tube, fast rotation, and spiral acquisitions, but they are limited to two or six slices (a 16-slice version also exists) per rotation. The XCT system of the Philips Brightview (Philips Healthcare, Milpitas, CA, USA) uses a flat panel detector, and data of one large (14 cm) axial field of view are acquired during a slow (typically 10 to 60 s) rotation in a fixed bed. Therefore, photon flux, scatter fraction, detector uniformity, and applied corrections (beam hardening, scatter, …) are likely to differ considerably between the four CT systems.

**Table 1:** Main characteristics of the four SPECT-CT systems used in the study

**Name**	**SPECT detector**	**NEMA spatial resolution**^**a**^**with LEHR collimator (mm)**				**CT**^**b**^
		**Planar**	**SPECT Central**	**SPECT Peripheral**		
				**Radial**	**Tangential**	
General Electric Discovery NM/CT 670	3/8 in. NaI crystal	7.4	≤9.9	≤9.9	≤7.5	80, 100, 120, and 140 kV
	59 PMT					10 to 440 (100) mA
	1 ADC/PMT					0.5, 0.6, 1.0, and 1.5 s rotation time (in spiral mode)
	40 × 54 cm FOV					24 rows - maximum 16 slices/rotation
General Electric Infinia Hawkeye 4	3/8 in. NaI crystal	7.4	≤9.9	≤9.9	≤7.5	120 and 140 kV
	59 PMT					1.0, 1.5, 2.0, and 2.5 mA
	1 ADC/PMT					2.0 or 2.6 rpm gantry rotation
	40 × 54 cm FOV					4 rows - 4 slices per rotation
Philips Brightview XCT	3/8 in. NaI crystal	7.4	≤10.3	≤10.5	≤9.0	120 kV
	59 PM					2.5, 5, 10, 15, and 20 mA
	1 ADC/PMT					12 s (fast) and 60 s (slow) rotation times
	40.6 × 54 cm FOV					CsI flat panel 14 cm axial FOV detector
Siemens Symbia T series	3/8 in. NaI crystal	7.4	≤11.4	≤11.7	≤8.4	80, 110, and 130 kV
	59 PMT					T2: 30 to 240 (100) mA; 0.8, 1.0, 1.5 s rotation time; 2 rows - 2 slices/rotation
	38.7 × 53.3 cm FOV					
						T6: 20–345 (100) mA; 0.6, 0.8, 1.0, 1.5 s rotation time; 16 rows - maximum 6 slices/rotation
						T16: 20 to 345 mA; 0.5, 0.6, 1.0, and 1.5 s rotation time; 24 rows - maximum 16 slices/rotation

^a^Full width at half maximum (at 100 mm for planar, with 15 cm rotation radius for SPECT) without scatter; ^b^underlined values are values used in this study. ADC, analog to digital converter; CT, computed tomography; FOV, field of view; NEMA, National Electrical Manufacturers Association, PMT, photomultiplier tube; SPECT, single-photon emission computed tomography.

**Table 2:** Main characteristics of the commercial 3D iterative reconstruction algorithms used in the study

**Name**	**Type**	**Corrections**			**Noise regularization**	**Manufacturer default number of**	
		**Attenuation**	**Scatter**	**Resolution**		**Subsets**	**Iterations**
General Electric Evolution for Bone	MAPEM	From CT data, bilinear conversion of HU into attenuation coefficients at 140 keV	Jaszczak’s dual energy window method with 115 to 125 keV scatter window	Matrix rotation	One-step late method with green prior and median root prior at last iteration	10	2
				Row convolution with spatial resolution kernel stored in look-up table			
Philips Astonish	OSEM	From CT data, HU segmentation using a step-like law, bilinear conversion of HU into attenuation coefficients at 100 keV, scaling to 140 keV	ESSE method	Convolution with spatial response function	Proprietary filtering (Hanning) of acquired projections and computed projections by forward-projection	15	2
Siemens Flash 3D	OSEM	From CT data, bilinear conversion of HU into attenuation coefficients at 140 keV	Modified triple energy window method with 108.5 to 129.5 keV scatter window	Matrix rotation	Gaussian post-filter (6-mm FWHM default value)	4	12
				Gaussian diffusion method with slabs			

3D, three-dimensional; CT, computed tomography; ESSE, effective source scatter estimation; FWHM, full width at half maximum; HU, Hounsfield units; MAPEM, maximum a posteriori expectation maximization; OSEM, ordered subset expectation maximization.

All three manufacturers use their own implementation of the bilinear law technique in order to convert the Hounsfield units (HU) of the CT images into linear attenuation coefficients for the SPECT photon energy. General Electric and Siemens use a bilinear transformation with scaling for the photon energy based on the pioneering work of Fleming [2]. Although we could not obtain detailed information, it is likely that the implementation by the two manufacturers is different. Philips uses a step-like curve for the conversion below 200 HU and a linear law above (Philips, personal communication). The resulting linear attenuation coefficients are for 100-keV photons, and a scaling to the SPECT photon energy is applied before reconstruction.

The approaches used for scatter corrections are also different. General Electric and Siemens use spectral-based corrections. In the General Electric cameras, a broad low energy window as suggested by Jaszczak [3] is used to evaluate the scatter contamination in the main energy window. Siemens scatter correction [4] is based on the triple energy window (TEW) method proposed by Ogawa [5] and refined by Ichihara [6]. However, the scatter is estimated from a unique lower energy window that is adjacent to the main window and has the same width, and not from the two very narrow energy windows of the original TEW. Contrary to Jaszczak’s or Ichihara’s original implementations, the scatter data are not subtracted from the main energy peak projections but are used in the iterative reconstruction loop. The effective source scatter estimation (ESSE) approach of Frey [7] is used by Philips [8]. This method is based on the density of the tissues traversed by the photons and on pre-computed convolution kernels that describe the degrading effect of scatter in matter on point source images. Tissue density is obtained from the CT images. This method is inherently linked to an iterative reconstruction with attenuation correction and seems to work best when the distance-dependent collimator response is included in the reconstruction algorithm [7]. The scatter estimate is computed at each iteration by the convolution of the actual image estimate with a spatially variant scatter kernel chosen on the basis of the tissue density and the source depth. All three methods are approximate and suffer from limitations [9-12].

Resolution recovery and 3D reconstruction considerably increase the computing load when compared to a simple 2D iterative reconstruction algorithm without resolution recovery. Therefore, to obtain with the actual computer reconstruction times acceptable in the clinical context, some accelerating schemes are used in addition to the well-known ordered subsets. The three manufacturers have made different choices. As an example of these differences, in Evolution and Flash3D, before any back- or forward-projection, the 3D image matrix is first rotated in a way that the transverse slices have rows parallel to and columns perpendicular to the camera detector.

As advocated by Wallis and Miller [13], the spatially variant camera resolution is taken into account both in the forward- and back-projection steps of all three algorithms. General Electric Evolution is based on the work done at the University of North Carolina and Johns Hopkins University [14,15]. After the matrix rotation, each row is convoluted with a kernel stored in look-up tables that describe the spatial response of the camera at this distance. Siemens Flash3D [4] makes use of the Gaussian diffusion method that, for a forward-projection, proceeds as follows. After the matrix rotation, the slice row farthest from the detector is convoluted with a Gaussian function that describes the difference in spatial resolution between this row and the immediately adjacent row. The result is added to the adjacent row, and the process is repeated. This means that the rows are convoluted with a succession of Gaussian functions that are not only sharper and sharper but also more and more intense. The steps are reversed in a back-projection. In order to proceed even faster, rows are grouped into so-called slabs [16], and the process described above is applied to these slabs and not to the individual rows. The higher the number of rows per slab, the faster is the reconstruction. However, this number should not exceed a few rows. Indeed, the resolution varies continuously with the distance from the detector, and this accelerated process applies an identical resolution to all rows of one slab. In Philips Astonish, the spatially variant camera response function is used in a convolution process at each forward- and back-projection step [8].

Noise regularization is also incorporated in all three algorithms. Flash3D [4] is basically an ordered subset expectation maximization (OSEM) algorithm without any particular noise regularization during the iterations. Noise control is performed after the last iteration with a Gaussian filter (post-filter) whose width can be changed by the user. The default value is 6 mm full width at half maximum (FWHM). Evolution is of maximum a posteriori expectation maximization (MAPEM) type. Noise regularization is obtained by applying a penalty to the image resulting from the previous iteration following the one-step late method introduced by Green [17]. At the last iteration, the regularization is based on the median root prior method that was first introduced for positron emission tomography (PET) [18] and whose extension to SPECT was shown to be straightforward [19]. In Astonish [8], a smoothing (Hanning) filter is applied to the acquired data (pre-filter) and after each forward-projection step. This proprietary noise regularization process is claimed to better preserve resolution than post-filtering. The filter is said to be matched as both the acquired and computed projections are filtered with the same filter.

All these developments have changed the 50-year-old Anger camera into an imaging system with quantitative potentialities comparable to those offered by PET, at least for static tomography imaging of one field of view. Indeed, SPECT large-field-of-view cameras are non-full ring systems, and the needed rotation for the detector(s) renders dynamic SPECT challenging [20]. Moreover, the need for a collimator implies a largely reduced sensitivity of SPECT compared to modern 3D PET. Nevertheless and despite these limitations, quantitative SPECT would be a highly valuable add-on to nuclear medicine in the context of radiotracers or radiotherapeutics development and of quantitative studies using well-established tracers.

The first approaches to quantitative SPECT ([21] and references therein, [22]) used systems that, with the exception of the General Electric Infinia Hawkeye, are no longer on the market. Moreover, some studies used separate stand-alone SPECT and CT systems, and the data were reconstructed with a locally developed software. More recently, Zeintl et al. [4] used an integrated SPECT-CT (Symbia T series) and its commercial 3D reconstruction (Flash3D) with attenuation, scatter, and resolution corrections. Knoll et al. [23] have focused on three image quality parameters, spatial resolution, contrast recovery, and background variability, of three modern SPECT-CT cameras, General Electric Infinia Hawkeye, Philips Brightview XCT, and Siemens Symbia T6, and their advanced 3D iterative reconstruction, General Electric Evolution for Bone, Philips Astonish, and Siemens Flash3D. Hughes et al. [24,25] compared General Electric Evolution for Bone, Philips Astonish, and Siemens Flash3D to their own reconstruction software. They used acquisitions of thorax phantoms performed with General Electric Infinia Hawkeye, Philips Precedence, and Siemens Symbia T6 SPECT-CT systems.

The aim of this study was to assess the quantitative capabilities of the four SPECT-CT systems available on the market from the three major vendors (General Electric Infinia Hawkeye and Discovery NM/CT 670, Philips Brightview XCT, and Siemens Symbia T series) together with their full 3D iterative reconstruction including attenuation and scatter corrections and resolution recovery (General Electric Evolution for Bone, Philips Astonish, and Siemens Flash3D). The accuracy of attenuation and scatter corrections was first investigated. The contrast recovery of small hot and cold regions was measured to obtain an estimate of the partial volume effect for small structures. The systems were calibrated using phantoms of different sizes to convert the counts per pixel into activity concentration (Bq/ml). Finally, the accuracy of the quantification was determined for the large uniform part of three different phantoms. Each correction step that leads to quantification in SPECT was therefore separately investigated. This allowed us to obtain firmer conclusions by excluding the constant possibility that different errors might be globally annihilated under the selected experimental conditions. All experiments were also designed in a way that they could be easily reproduced by other investigators in similar or different systems at a low cost.

## Methods

### SPECT-CT cameras

Four state-of-the-art SPECT-CT cameras were tested (Table 1): General Electric Discovery NM/CT 670 (Discovery) and Infinia Hawkeye 4 (Infinia), Philips Brightview XCT (Brightview), and Siemens Symbia T6 (Symbia). Some experiments were repeated on a second Philips Brightview XCT and a Siemens Symbia T2.

### Activity measurement

All activities were carefully measured with the radionuclide calibrator available in the department, and the time of measurements was recorded. These radionuclide calibrators undergo a daily quality control following the Belgian Hospital Physicist Association and Federal Agency for Nuclear Control protocol.

### Attenuation and scatter correction accuracy

To assess for attenuation and scatter correction, the National Electrical Manufacturers Association (NEMA) NU2-1994 phantom was used with air, water, and Teflon cold inserts (Table 3) and 740 MBq of ^99m^Tc in the background. The acquisition was set up so that the first projection contained 880 kcounts. An analysis similar to the NEMA NU2-1994 method was conducted as follows: a region of interest (ROI) of 30-mm diameter and 180-mm height was drawn on each insert, and eight ROIs of the same dimensions were drawn in the background. The residual fraction (RF) in the cold inserts was calculated as RF = *C*_insert_ / *C*_background_, with *C*_insert_ and *C*_background_ being the mean number of counts per pixel in the insert and the background, respectively. The NEMA phantom was also imaged with a second Brightview camera and a Symbia T2. A shorter acquisition resulting in four times fewer total counts was performed with the Siemens T6 system.

**Table 3:** Main characteristics of the cylindrical phantoms used in the study

**Name**	**Height (mm)**	**Diameter (mm)**	**Initial activity (MBq)**	**Counts in first projection (kcounts)**
Contrast	300	200	740 ± 45	880
L	80	94	300 ± 18	670
M	80	54	300 ± 18	220
NEMA	190	200	740 ± 45	880
S	80	16	300 ± 18	20
XL	300	200	740 ± 45	880

Contrast, cylindrical phantom with uniform cold and hot rods parts. L, cylindrical phantom of 8-cm height and 9.4-cm diameter; M, cylindrical phantom of 8-cm height and 5.4-cm diameter; NEMA, National Electrical Manufacturers Association NU2-1994 phantom; S, cylindrical phantom of 8-cm height and 1.6-cm diameter; XL, cylindrical phantom of 30-cm height and 20-cm diameter.

### Contrast recovery

The contrast recovery was investigated with a cylindrical phantom (Table 3) containing three main parts. The first part was a uniform compartment of 65-mm height. The two other parts were made of 85-mm-high rods. One contained seven cold rods (with diameters of 6, 8, 10, 12, 16, 20, and 25 mm), and the other contained seven hot rods (with diameters of 4, 6, 8, 10, 13, 16, and 20 mm). For each set of rods, the largest rod was on the phantom axis, and the other six were uniformly distributed at 5 cm from the axis. The activity in the phantom was 740 MBq of ^99m^Tc, and the acquisition was set up so that the first projection contained 880 kcounts. As the radioactive liquid circulated freely between the phantom compartments, a thorough shaking after the ^99m^Tc injection ensured an identical activity per volume unit in the uniform part, the hot rods, and the background of the cold rods.

A cylindrical ROI with a diameter equal to 80% of the physical diameter of the phantom and 24 mm in height was drawn on the uniform part of the phantom. Two different cylindrical ROIs were drawn on the rods, the first with a diameter equal to the physical diameter of the rod (full ROI) and the second with half its diameter (half ROI). The height of the ROI corresponded to 11 slices (29.15 ± 1.65 mm), and the ROI was centered on the rod. The contrast recovery coefficient (CRC) was calculated for both ROIs with the uniform part as reference as follows:

Hot inserts: CRC = *C*_rod_ / *C*_uniform_

Cold inserts: CRC = 1 - (Crod/Cuniform)

with *C*_rod_ and *C*_uniform_ being the number of counts per pixel in the insert and the uniform part, respectively.

With the Symbia T6, the contrast phantom was scanned three times successively on the same day with the same acquisition parameters to assess the repeatability, and three identical acquisitions spaced by several weeks were performed to check the reproducibility. This phantom was also scanned with the Symbia T2, always with the same acquisition parameters.

### Thyroid phantom

To image structures other than cylinders, the well-known Picker’s thyroid phantom [26] was imaged. The activity was 200 ± 12 MBq of ^99m^Tc, and the acquisition was set up so that the first projection contained 220 kcounts. The phantom was lying in air on the camera bed, mimicking the thyroid anatomical position of a patient in supine position. There was no additional background and no scattering media added. This phantom could not be scanned with Discovery due to a technical problem at the time we had access to this system.

### Quantification

The counts per volume unit to activity concentration (Bq/ml) conversion factor (CF) was determined using three phantoms of different sizes, namely XL (cylindrical, height 30 cm, and diameter 20 cm), L (cylindrical, height 8 cm, and diameter 9.4 cm), and M (cylindrical, height 8 cm, and diameter 5.4 cm) phantoms (Table 3). The activities were 740 MBq for XL and 300 MBq for L and M, and the acquisitions were set up so that the first projection contained 880 kcounts for XL, 670 kcounts for L, and 220 kcounts for M.

On each phantom, seven cylindrical ROIs were drawn to obtain a conversion factor CF(phantom,%ROI) associated with the phantom and the ROI used. All ROIs had a height equal to 85% of the phantom’s physical height, and their diameter varied between 60% and 100% of the phantom’s physical diameter.

For each ROI, the mean count per milliliter was divided by the acquisition time of a projection to obtain the count rate per unit volume (counts/ml/min or cpm/ml). For the Siemens Flash3D reconstruction software, no decay correction was available, and the count rate was first corrected for ^99m^Tc radioactive decay as suggested by Zeintl et al. [4]. For the other types of software, the decay correction option was used. CF(phantom,%ROI) (cpm/ml/MBq/ml) was then determined by dividing the count rate per unit volume (cpm/ml) by the activity concentration decay corrected at the beginning of the acquisition. The CFs were calculated for the data reconstructed with eight subsets and 24 iterations. A total of 21 different CFs were obtained for the three phantoms. There were seven CFs per phantom, each corresponding to an ROI.

Using these 21 CFs, the reconstructed activity in the uniform parts of the NEMA and contrast phantoms and on a small uniform cylinder S (Table 3) was computed. This small phantom was used to assess the feasibility of using a marker (i.e., an object of known activity) during a patient examination. The activity in the S phantom was about 300 MBq, and the time per projection was such that the first projection contained 20 kcounts. Reconstructions were performed with eight subsets and 24 iterations. For the NEMA and contrast phantoms, the background or the uniform ROI described above was used. The mean count rate per milliliter in the ROI was converted into becquerel per milliliter with the CF(phantom,%ROI). For the S phantom, a ROI was drawn at a level of 1% of the maximum value to account for all the activity in the object [21]. The count rate per milliliter was evaluated by dividing the total number of counts per minute by the volume of the phantom, and finally, the CFs were applied to obtain the activity concentration (Bq/ml). Three sets of seven reconstructed activities were obtained in this way for each phantom (NEMA, contrast, and S). Each set corresponded to a different calibration phantom (M, L, and XL), and the seven different activities of one set corresponded to the seven ROIs drawn on one particular calibration phantom. The M phantom was scanned with the Symbia T6 and identical acquisition parameters three times successively on the same day, then 3 weeks later, and finally 10 months later in order to assess the repeatability and reproducibility of the CF determination procedure.

### Acquisitions

All images were acquired in H-mode with LEHR collimators on a 360° (180°/head) orbit. A total of 128 (Philips and Siemens) or 120 (General Electric) projections were acquired in a 128 × 128 pixel matrix with a hardware zoom chosen so that the pixel size was 2.65 ± 0.15 mm. The trajectory was circular with a radius of 25 cm. This value was found to be the joint smallest possible radius for the four cameras when the bed was in the field of view and the patient collision safety system was in use. On the Symbia T6, data of the M, L, and XL uniform phantoms were also acquired with the body contour option that led to an elliptical orbit whose minor axis was oriented in the anterior-posterior direction. The standard system energy window for ^99m^Tc was used. It was set at 140 keV with a total width of 20% for the General Electric and Philips cameras, and 15% for the Siemens cameras. The contrast and NEMA phantoms were centered in the field of view; the S, M, L, and XL phantoms were axially offset by about 10 cm.

For attenuation correction, a standard CT protocol was used (Table 1). As its CT component is non-conventional but a flat panel system with slow rotation, two different CT protocols (Table 1) were used with the Philips Brightview XCT: a low tube current was applied with the so-called fast protocol, which is recommended for SPECT attenuation correction, and a high tube current was applied with the slow protocol, which is recommended for diagnostic CT procedures.

### Reconstructions

The camera manufacturers’ 3D iterative reconstruction with attenuation and scatter correction and resolution recovery was used with eight subsets and various numbers of iterations, namely Philips Astonish for Brightview, General Electric Evolution for Bone for Discovery and Infinia, and Siemens Flash3D for Symbia. The manufacturers’ default parameters for attenuation and scatter corrections and for contrast recovery were systematically used. Flash3D reconstructions included a post-smoothing as recommended by Siemens. Unless otherwise notified, the default 6-mm FWHM Gaussian filter was used. NEMA and contrast phantom data were also reconstructed without performing the scatter correction. For some Siemens Symbia T6 data, Siemens 2D OSEM reconstruction was also performed with eight subsets. This reconstruction applies resolution recovery only in the transverse direction and does not perform scatter correction.

### Processing software

A Medical Image Data Examiner (AMIDE, version 0.8.19; Andy Loening) freeware running on a Macintosh (Apple) laptop computer was used to process the reconstructed data. The ROIs were first drawn on the CT images and reported afterwards on the SPECT images. The AMIDE-dedicated tool was used to obtain the total number of counts and, when needed, the total number of pixels in an ROI on a SPECT image or the mean attenuation coefficient value in an ROI on a CT image.

## Results

### Attenuation and scatter correction accuracy

**Figure 1:**
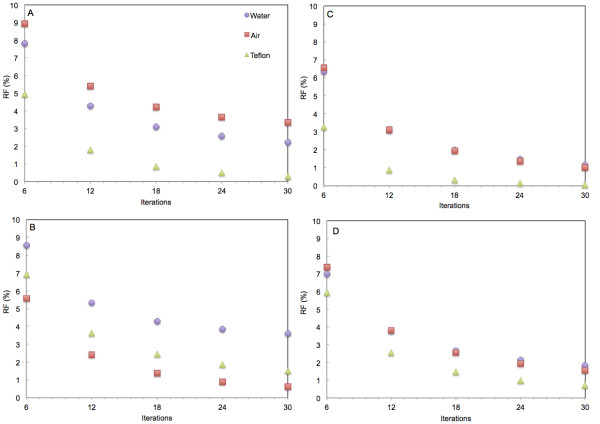
**Residual fraction in cold inserts of the NEMA NU 2–1994 phantom.** Results for the three non-emitting air (square), water (circle), and Teflon (triangle) inserts as a function of the number of iterations with eight subsets for the four SPECT-CT systems. (**A**) Philips Brightview XCT. (**B**) General Electric Discovery NM/CT 670. (**C**) General Electric Hawkeye 4. (**D**) Siemens Symbia T6. All reconstructions with attenuation and scatter corrections and resolution recovery.

The residual fraction in the three inserts of the NEMA phantom decreased with the number of iterations (Figure 1). After six iterations, residual fractions were in the range 5% to 9% (3% to 7% for Infinia). At 30 iterations, all three residual fractions were below 1% for Infinia, 2% for Symbia, 3% for Brightview, and 3.5% for Discovery. Residual fraction in Teflon was around 1.5% for Discovery and below 1% for all other systems. In air, it was below 1% for both General Electric systems and around 1.5% for Symbia or 3% for Brightview. The residual fraction in water was around 1% for Infinia, 2% for Symbia, 2.5% for Brightview, and 3.5% for Discovery. When scatter was not corrected for (Additional file 1), residual fractions in the water and Teflon inserts fluctuated with the number of iterations between 9% and 12% for Infinia and Symbia, between 9% and 14% for Brightview, and between 14% and 15% for Discovery. In the air insert (Additional file 1), the residual fraction decreased with the number of iterations for all four systems, from 5% to 6% at six iterations to less than 1.5% at 30 iterations for all systems.

The linear attenuation coefficient of water was very close to the theoretical value (0.153/cm at 140 keV [27]). However, for Teflon, it was lower than the theoretical value (0.301/cm at 140 keV [27]) by about 10% for Brightview, 14% for Infinia, 22% for Symbia, and 30% for Discovery. Air data are not reported in detail; this point is further discussed in the ‘Discussion’ section. For Brightview cameras, using the CT fast protocol with low tube current or the CT slow protocol with high tube current resulted in almost identical residual fraction and attenuation coefficient values.

The NEMA phantom was also imaged with a second Brightview camera and a Symbia T2. Residual fraction values were almost identical with an absolute difference less than 1% between the two Brightview systems and less than 2.5% between the Symbia T6 and T2. Attenuation coefficients obtained with the two Brightview or the two Symbia systems were identical in water and differed by less than 3% in Teflon. Larger variations were observed in air. A shorter acquisition of the NEMA phantom resulting in four times less total counts was also performed with the Siemens T6 system. The values for the residual fractions in the inserts differed from those obtained with the high count acquisition by less than 0.5%.

### Contrasts

The NEMA planar spatial resolution of the four SPECT cameras appeared identical, whereas their NEMA SPECT spatial resolutions were found different (Table 1). A preliminary study (Additional file 2) used FBP and Chang attenuation correction to reconstruct the data obtained in the present study. The contrast recovery coefficients were determined using an identical procedure and were compared to those obtained over the past 5 years in the same scanning conditions with eight different dual-head stand-alone SPECT cameras. It was observed that the four SPECT-CT systems under investigation did not behave differently from the older systems under the acquisition conditions chosen for this study. Moreover, the four SPECT-CT cameras delivered similar contrast recovery coefficients for the images obtained with FBP reconstruction.

**Figure 2:**
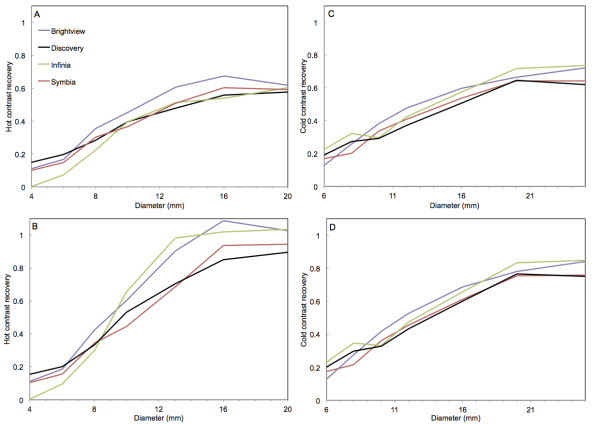
**Contrast recoveries for the hot and cold rods of the contrast phantom.** Values obtained for the four SPECT-CT systems after 30 iterations with eight subsets. (**A**, **B**) Hot rods. (**C**, **D**) Cold rods. (**A**, **C**) Full ROI. (**B**,** D**) Half ROI.

With the 3D iterative reconstructions, hot and cold contrasts increased with the number of iterations (Additional files 3, 4, 5, and 6). For all algorithms, contrast recovery started to level off around 24 iterations. These plateau contrast recoveries are illustrated in Figure 2. The highest contrasts were observed from 16-mm hot rod diameter or 20-mm (25 mm for Brightview) cold rod diameter. With the full ROI, these hot contrast recoveries (mean of 16- and 20-mm rod values) were 0.56 for Discovery, 0.60 for Infinia and Symbia, and 0.67 for Brightview. With the half ROI, they increased to 0.85 for Discovery, 0.95 for Symbia, 1.01 for Infinia, and 1.10 for Brightview. With the full ROI, the maximum cold contrast recoveries (mean of 20- and 25-mm rod values) were 0.66 for Discovery and Symbia and 0.74 for Brightview and Infinia. With the half ROI, values were 0.78 for Discovery and Symbia and 0.86 for Brightview and Infinia.

When scatter correction was not performed, contrast recoveries were generally lower. The reduction usually amounted to between 0.05 and 0.1 for the two largest hot rods. However, for the half ROI, the decrease was 0.25 for Infinia and 0.3 for Brightview. For the two largest cold rods and whatever the ROI, the contrast decreased by 0.2 for Infinia and by approximately 0.1 for the three other systems. The smaller the hot or cold rod size, the smaller is the difference in contrast recovery between images corrected and not corrected for scatter. For both Brightview cameras, the scatter correction almost did not change the contrast recovery of the two smallest (≤6 mm) hot rods, whereas higher contrast recovery was observed for the non-scatter-corrected images of the three other systems. Differences were between 0.05 and 0.2. For Infinia (Figure 2), the contrast recovery of the 4-mm rod obtained with scatter correction was almost 0.

When the data were not post-filtered in Flash3D (Additional file 7), contrast recovery increased very modestly (less than 0.05) for the cold rods but more severely for the hot rods. For the larger ones, the increase was as large as 0.2 in the half ROI, and the contrast recovery slightly exceeded 1.2. Using the CT fast protocol with low tube current or the CT slow protocol with high tube current on the Brightview XCT cameras resulted in almost identical contrast recovery values.

The contrast phantom was also scanned with a Symbia T2 system and reconstructed with Flash3D (with all corrections and a post-filter). The contrast recoveries generally differed by less than 0.05 from those recorded with Symbia T6. However, larger differences rising to 0.1 to 0.2 were observed for the largest hot rods, especially with the half ROI, and the contrast recovery of the smallest hot rod was almost 0.

### Quantification

**Figure 3:**
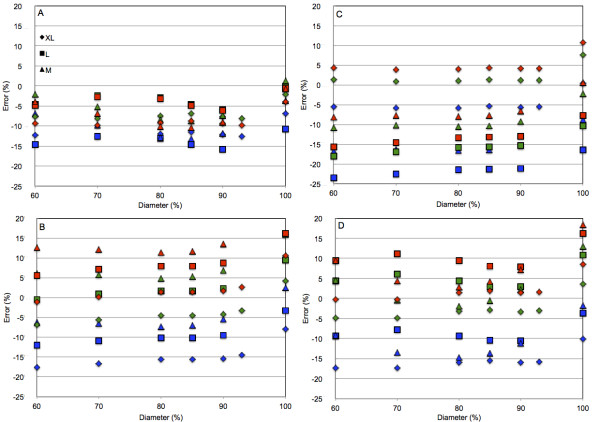
**Quantification error obtained for the three calibration phantoms and the three test phantoms.** The error is plotted as a function of the calibration phantom ROI diameter expressed as a percentage of the phantom physical diameter for the four SPECT-CT systems. The quantification was performed for the uniform part of the contrast (red) or NEMA NU 2–1994 attenuation and scatter correction accuracy (green) phantom and the S phantom (blue) using the conversion factor obtained with the M (triangle), L (square), or XL (diamond) phantom. All reconstructions with 24 iterations and eight subsets. (**A**) Philips Brightview XCT. (**B**) General Electric Discovery NM/CT 670. (**C**) General Electric Hawkeye 4. (**D**) Siemens Symbia T6.

The error in reconstructed activity per volume unit in the uniform part of the contrast and NEMA phantoms and the total reconstructed activity in the S phantom are presented in Figure 3 as a function of the diameter size (expressed as a percentage of the physical diameter size) of the cylindrical ROI drawn on the three (M, L, and XL) calibration phantoms. It appeared that the activities obtained using 100%-diameter-size ROIs were systematically higher by at least 5% than the activities obtained with the 60%- to 90%-diameter-size ROIs. Between 60% and 90% of the physical diameter size, the reconstructed activities almost did not depend on the ROI diameter size for the XL phantom, but variations were observed for the L and M phantoms, and the variations were the largest for the smallest phantom. The reconstructed activities depended clearly on the calibration phantom with differences between the phantoms starting at a low level (but within expected measurement errors), 2% to 3% for Brightview and increasing to as much as 15% to 20% for Infinia between the L and XL phantoms. For Discovery, the highest activities were obtained with the M phantom, and the lowest with the XL phantom. For Infinia, the highest activities were recorded for the XL, and the lowest with the L phantom; the situation was reversed for Symbia. Table 4 summarizes the mean quantification errors for the ROI with a diameter of 60% to 90% of the physical diameter size, the three test phantoms, and the three calibration phantoms.

**Table 4:** Mean quantification error for 60% to 90% ROI diameter of the calibration phantom physical diameter

**SPECT-CT systems**	**Quantification error (%)**								
	**Contrast**			**NEMA**			**S**		
	**XL**	**L**	**M**	**XL**	**L**	**M**	**XL**	**L**	**M**
Brightview	−9.29	−4.34	−8.08	−6.32	−4.05	−6.32	−12.08	−14.15	−10.91
Discovery	0.72	7.48	12.27	−5.12	1.25	5.76	−16.15	−10.52	−6.54
Infinia	4.11	−13.95	−7.64	1.17	−16.38	−10.25	−5.66	−22.02	−16.31
Symbia	0.82	9.22	5.62	−3.83	4.18	0.76	−16.45	−9.49	−12.47

Results are presented for the four SPECT-CT systems, the three calibration phantoms (XL, L, and M), and the three test phantoms (Contrast, NEMA, and S).

For three successive scans of the M phantom with Symbia T6, the CFs differed by less than 0.5% for all ROIs. CF values determined 3 weeks later were 3.4 ± 0.1% higher. After a 10-month delay, the CFs had again slightly increased. The increase was the lowest (5%) for the largest ROI, 6.6% for the 70% ROI, and rose to 13.6% for the 60% ROI.

### Artifacts

**Figure 4:**
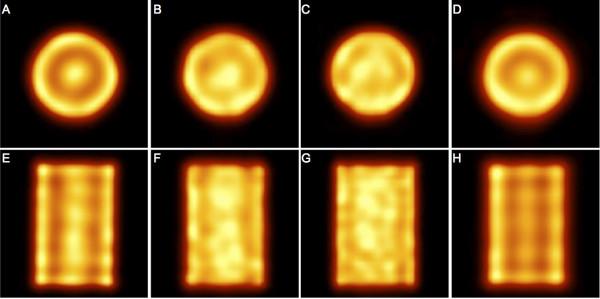
**Transverse and coronal slices of the M phantom obtained after 24 iterations.** (**A**, **E**) Philips Brightview XCT and Astonish. (**B**, **F**) General Electric Discovery NM/CT 670 and Evolution for Bone. (**C**, **G**) General Electric Hawkeye 4 and Evolution for Bone. (**D**, **H**) Siemens Symbia T6 and Flash3D. All are reconstructions with eight subsets. Hot-iron color scale from 0% to 110% of slice maximum.

**Figure 5:**
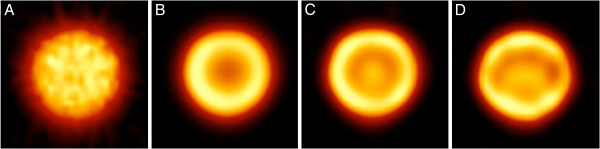
**Transverse reconstructed slices of the M phantom imaged with the Symbia T6.** (**A**, **B**, **C**) Circular orbit. (**D**) Non-circular orbit. (**A**) FBP. (**B**) Flash3D and six iterations. (**C**, **D**) Flash3D and 24 iterations. All are reconstructions with eight subsets. Hot-iron color scale from 0% to 110% of slice maximum.

**Figure 6:**
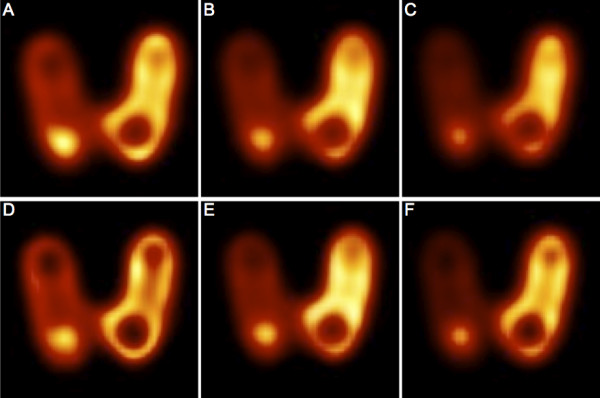
**Coronal reconstructed slices of the Picker’s thyroid phantom.** (**A**, **B**, **C**) Six iterations. (**D**, **E**, **F**) 24 iterations. (**A**, **D**) Philips Brightview XCT and Astonish. (**B**, **E**) General Electric Infinia Hawkeye 4 and Evolution for Bone. (**C**, **F**) Siemens Symbia T6 and Flash3D. All are reconstructions with eight subsets. Hot-iron color scale from 0% to 110% of slice maximum.

**Figure 7:**
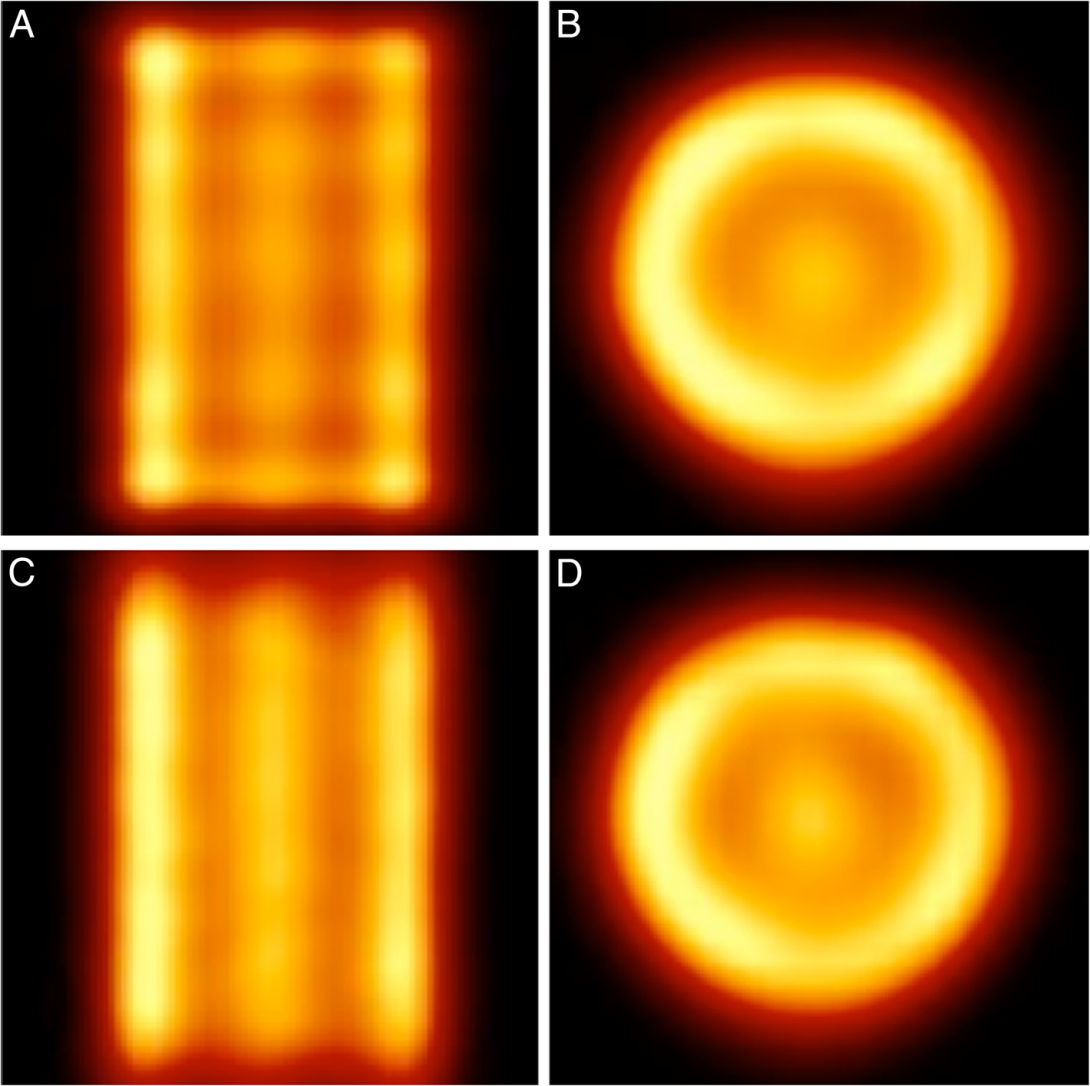
**Coronal and transverse reconstructed slices of the M phantom imaged with Symbia T6.** (**A**, **B**) Siemens Flash3D. (**C**, **D**) Siemens OSEM 2D with resolution recovery in the transverse plane. All are reconstructions with eight subsets and 24 iterations. Hot-iron color scale from 0% to 110% of slice maximum.

Figure 4 presents some slices of the M phantom. Edge artifacts were observed with all phantoms (some images of other phantoms are presented in Additional files 8 and 9) and with all four systems, but the artifact intensity was system-dependent and clearly higher on the Brightview and Symbia images than on the two General Electric cameras. The shape of the edge artifact was different when the head orbit was elliptical instead of circular (Figure 5). Oscillation artifacts were also clearly visible on the Brightview and Symbia images. These oscillation artifacts changed with the number of iterations (Figure 5). The artifacts were also observed on non-cylindrical phantoms, as demonstrated by the images of the thyroid phantom presented in Figure 6. It is worth emphasizing the usefulness of a high number of iterations for distinguishing the small cold nodule in the left lobe. The Siemens software allows the reconstruction of the data with a 2D OSEM algorithm that includes resolution recovery in the transverse planes but not in the axial direction. As illustrated in Figure 7, the edge and oscillation artifacts in the axial direction were not observed when using this reconstruction strategy. Circular-shaped artifacts were clearly visible on transverse slices obtained with the two General Electric cameras ( Additional file 8). These artifacts were not observed when the data were reconstructed with FBP or with OSEM including attenuation and scatter corrections but not resolution recovery. These artifacts corresponded to circles centered in the reconstructed field of view.

## Discussion

Absolute quantification of SPECT images is an old dream but became clinically feasible only very recently, thanks to the introduction of commercial systems which combine SPECT-CT technology and fast 3D reconstruction algorithms with attenuation and scatter corrections and resolution recovery. In this study, we have considered the General Electric Infinia Hawkeye 4, Philips Brightview XCT, and Siemens Symbia T6 SPECT-CT cameras, which have been on the market for several years, but we also looked at the very recently introduced General Electric Discovery NM/CT 670. The manufacturers’ 3D iterative reconstruction with attenuation, scatter, and resolution corrections was systematically used. The manufacturers’ default parameters for these corrections were systematically used while the impact of the number of iterations was studied. Attenuation and scatter accuracy, contrast recovery of hot and cold regions of different sizes, and finally, quantification using three calibration phantoms of different sizes have been analyzed. To the best of our knowledge, this is the first homogeneous comparative study between the four state-of-the-art SPECT-CT systems of three major nuclear medicine vendors.

### Attenuation and scatter correction accuracy

The first step in the quantification of nuclear medicine images is clearly a correction for the attenuation and scatter of the emitted photons [9,10]. It was therefore worthwhile to first assess the accuracy of the attenuation and scatter corrections applied in the four systems. For that purpose, the NEMA NU2-1994 methodology was adopted. Although primarily developed for PET, this methodology is perfectly applicable to SPECT. In contrast to PET reconstructions, the SPECT manufacturer's 3D reconstructions did not generally allow reconstruction with calculated attenuation correction (the Chang method, for example). Therefore, only the combined attenuation and scatter correction accuracy could be evaluated.

The use of Teflon insert could be questioned. Indeed, it has been demonstrated with PET-CT that the HU conversion laws used for low-density material and bone does not fully apply to Teflon [28]. This is mainly due to the large differences in physical effect leading to photon attenuation. Indeed, the photoelectric effect, together with Compton scattering, contributes to X-ray photon attenuation, whereas 511-keV photon attenuation almost results from Compton scatter alone. However, attenuation correction using CT data and bilinear conversion of HU in linear attenuation coefficients have been largely validated for PET-CT, and non-biological materials are increasingly present in scanned patients. In this sense, the use of the Teflon insert was not considered as a limitation of the study but merely as an add-on. For example, Shcherbinin et al. [21] also used a Teflon insert to mimic the lumbar spine in their investigation of the quantitative potentialities of Infinia Hawkeye 4.

The air, water, and Teflon inserts of the NEMA NU2-1994 phantom are cold compartments. When scatter correction was applied (Figure 1), the residual fractions decreased with the increase of the number of iterations and reached values below 4% at 30 iterations for all systems. Without scatter correction (Additional file 1), the residual fraction in water and Teflon remained stable after about ten iterations but still continued to decrease in the air insert. Scattering in air is expected to be very low, and therefore, the air insert should approximately correspond to a perfect cold region, whether the scatter correction is being applied or not. On the contrary, the more dense water and Teflon inserts should only behave as a perfect cold region when the scatter is corrected for. Convergence of iterative reconstructions is known to depend on the local contrast and is expected to be the slowest for the coldest regions. This is exactly what is observed in air with or without the scatter correction and in the two more dense media when scatter correction is applied.

Without scatter correction, residual fractions in water and Teflon were system-dependent, with differences of up to 5% between Infinia and Discovery. For Brightview, the residual fraction in air was even higher with scatter correction (RF ≈ 3%) than without the correction (RF ≈ 1%). Differences in scatter contamination between camera models have recently been reported in a multi-centric study [29]. The most striking conclusion nevertheless is that despite the use of three different scatter correction techniques, all the systems achieved, in the three cold inserts of the NEMA NU2-1994 phantom, approximately identical and very low (≤4%) residual fractions at 30 iterations.

The linear attenuation coefficients were very close to the expected value for water but were generally lower than the expected value for Teflon. As already mentioned above, this could result from the HU conversion laws that are tailored to biological materials. The under-correction for attenuation of Teflon could explain the lower fractional residues observed in this insert. The values of the air linear attenuation coefficient are not reported in detail. They ranged from 0.0000001/cm (Brightview) to 0.001/cm (Symbia). It is evident that small differences in HU calibration and/or the difference between the HU conversion laws used can lead to large differences in the measured value of the very low air linear attenuation coefficient. However, the values are so low that the attenuation correction is almost not affected by their accuracy. For Brightview, the CT protocol (fast or slow, high or low current) seemed to not influence the results, at least for a phantom with the size and the composition of the NEMA NU2-1994 attenuation and scatter accuracy phantom.

### Contrasts

The contrast part of this study was conducted to obtain an estimation of the object size below which quantification would unavoidably be corrupted by the partial volume effect. The use of rods for assessment of contrast recovery with 3D reconstructions could be questioned. A sphere phantom was considered unpractical in the context of the present study performed on six systems belonging to five different departments and with some time limitation in camera availability. Indeed, a sphere phantom is much more fragile than a rod phantom, and the filling procedure is clearly longer. Moreover, the experiment would have been repeated at least two to three times to keep the noise variability sufficient low. The rod phantom allowed the summing of the results obtained in several slices which, combined with a high number of acquired counts, helped to reduce noise variability. A definite advantage of the contrast phantom is its ease and low cost of manufacture. The contrast recovery coefficients obtained with a sphere phantom would depend on sphere and background activities, sphere to background contrasts, and the number of total acquired counts [23]. The rods offer the opportunity for infinite contrast, and this could be seen as a very favorable aspect. It is expected that contrast recovery in a clinical context would be different and presumably lower. Therefore, the contrast recovery coefficients obtained in this study represent an upper limit.

The use of the circular trajectory with a 25 cm radius could also be questioned. In the clinical context, the automatic body contour device is generally activated, and this results in non-circular trajectories with a variable distance between the axis of rotation and the camera heads. For some slim patients and some explorations, this distance would be less than 25 cm, especially when the camera heads are in imaging positions close to the anterior-posterior direction. However, for many other cases (trunk explorations and obese patients), this distance would also be longer for all head positions. The selection of the joint smallest possible radius for the four cameras was found to be an acceptable compromise. Moreover, the circular trajectory with a manually fixed radius renders our experiments very easy to repeat on other already existing (for example the SPECT-CT system from another manufacturer) or future SPECT-CT systems.

Using the manufacturer’s 3D iterative reconstructions, hot and cold contrast recovery improved with the number of iterations (Additional files 3,4,5, and 6). However, above 24 iterations, the improvements were only marginal, and 30 iterations was chosen as the end point of this study. This was justified by the fact that the noise level steadily increased with the number of iterations (data not shown), while it is always desirable to keep this level as low as possible. The contrast recovery increased with the rod diameter. Whatever the ROI size used to evaluate the contrast, the hot contrast saturated when the rod diameter reached 16 mm. The cold contrast of the General Electric and Siemens cameras saturated for a rod diameter of 20 mm and above, but no saturation could be clearly observed with Brightview. It should be emphasized that the data for the largest hot or cold rod should be taken with some caution. Indeed, this rod is located on the phantom axis, and the phantom was centered in the field of view. Therefore, this rod is more prone to uniformity artifacts than the six other peripheral rods [30-32]. Moreover, the image resolution was demonstrated not to be isotropic, although resolution recovery is included in the reconstruction algorithm [23]. Maximum contrast recovery was slightly system-dependent. With the half ROI, it was in the range 0.85 to 1.1 for the largest hot rods and in the range 0.78 to 0.86 for the largest cold rods. These values were generally lower when scatter was not corrected for and the amount of reduction was system-dependent. However, with the exception of Brightview, the contrast recovery of the two smallest hot rods was found to be higher when scatter correction was not applied. For Infinia and the Symbia T2, the contrast recovery of the smallest (4 mm) hot rod dropped to 0 in scatter-corrected images. This agreed with the observation that the scatter contamination and the performance of the scatter correction varied between the four systems. In the clinical context, the use of scatter correction with a resulting decreased hot contrast for small structures is questionable. For this contrast phantom, the CT protocol (fast or slow, high or low current) used with Brightview had no influence on the results.

Thanks to their resolution recovery, the three reconstruction algorithms delivered images with improved contrast for the small structures. Nevertheless, for accurate quantification, some strategy for partial volume correction remains necessary. The lower contrast recoveries observed for the full ROI as compared to the half ROI show that the partial volume effect remains present. Moreover, although the contrast recovery for the largest hot rods approached unity with the half ROI, they were not all equal to 1, and some differed from 1 by values as large as 0.15 (Figure 2). This indicates that the partial volume correction technique should be tailored to the particular SPECT system and reconstruction algorithm used. Moreover, the reconstruction artifacts should also be considered in the framework of accurate quantification.

### Artifacts

Edge and noise artifacts in maximum likelihood reconstructions have been observed and studied for a long time [13,14,33]. Noise was said to result from maximum likelihood expectation maximization (MLEM) doing a too good job [33]: ‘MLEM is so successful in producing images that are consistent with the acquired data that the noise is also fully reproduced.’ Edge artifacts seemed to result from the impossibility to recover frequencies whose amplitudes are too low [33]. Therefore, the frequency content of the images is incomplete. This becomes dramatic at edges where representations are made of a very wide frequency range (infinite range for a sharp edge) and result in the observed overshoots [33]. The link between the edge and oscillation artifacts seems not to have been clearly established. However, it was observed that techniques tailored to reduce or suppress the edge artifacts also reduced or suppressed the oscillation artifacts [33].

Edge and oscillation artifacts were observed with all phantoms, whatever their shape and with all four systems (Figures 4 and 6, and Additional files 8 and 9). Ringing artifacts were already observed by Vija et al. in their early study of Flash3D [34]. The artifact intensities appeared to be system- and phantom-dependent. For the two General Electric cameras, uniformity artifacts were also present, and they could have obscured some other artifacts. It is very interesting to note that the uniformity artifacts were not observed when the images were reconstructed with FBP or 2D OSEM (without resolution compensation). This indicates that the use of reconstruction algorithms with resolution recovery implies a revision of the acquisition parameters, and particularly the total number of acquired counts, of the procedures used to generate the uniformity correction matrix. As an example, Vija et al. [34] mentioned the use of very-high-count (up to 0.8 billion) floods for uniformity correction of data reconstructed with Flash3D. With Symbia T6, a few SPECT acquisitions of the uniform phantoms were conducted with an elliptical orbit in addition to the circular orbit, and the edge ring artifact was elliptically shaped (Figure 5). The Siemens software allows 2D OSEM reconstructions with resolution recovery only in the transverse plane (no resolution recovery is in this case performed in the axial direction). On these 2D OSEM reconstructed coronal and sagittal slices, the stripes perpendicular to the rotation axis that were clearly visible on the Flash3D reconstructed images were not observed (Figure 7).

In a small structure, the edges come very close to each other, and the edge artifacts collapse. This results in a too-high activity in the central area, and the structure could appear smaller on the nuclear medicine image than on the structural image, as illustrated in Additional file 9. One other issue for iterative reconstruction is the inability to measure the resolution obtained using point or line source in air [13] and the preferable usage of a contrast phantom to evaluate the performance in distinguishing between objects of different contrasts [35].

The regularization step included in the reconstruction algorithm should have some control on the overshoot of small structures. As part of the iterative loop, this step could not be deactivated in Astonish or Evolution. However, Flash3D allowed the bypassing of the post-filter. Without this final smoothing, cold contrast recovery was only modestly increased, but the increase was much more important for the hot contrast recovery, and values largely above 1 were observed (Additional file 7). A detailed study with different structures, count statistics, and pixel sizes would probably help to fine-tune the post-filter of Flash3D in order to optimize the compromise between contrast recovery and edge artifacts for various acquisition and reconstruction parameters. Such a study was beyond the scope of this work.

### Number of iterations

When ordered subsets are used, the number of subsets has to be considered together with the number of iterations. Generally, one uses the product of both, the so-called number of equivalent number of MLEM iterations (MLEMit). All results demonstrated the need for a sufficiently high number (24 × 8 or 192) of MLEMit to obtain convergence of the iterative algorithm and efficient scatter correction or maximum contrast recovery. This number greatly exceeds the default setting of all three manufacturers, which ranges from 20 MLEMit to 48 MLEMit (Table 2). These settings seem to have been chosen with the main aim of generating images with spatial resolution similar to FBP or OSEM but with a lower noise content and allowing a reduction in scan time and/or patient dose [23]. In the framework of quantification, convergence of the iterative algorithm in all regions of the image is mandatory. We therefore decided to select 24 iterations with eight subsets for our study of the quantification. The small cold nodule in the thyroid phantom highlights the usefulness of a high number of iterations in clinical routine.

### Quantification

Quantification requires the conversion of the recorded counts per pixel into activity per volume unit. This is usually obtained through a calibration step where a source of known activity is scanned. One study has presented the use of a point source and of planar acquisitions to obtain the conversion factor [21]. However, most of the other studies copied the extensively validated PET procedure where a large source of known activity and volume are scanned [4,21]. This last methodology was adopted in this work, but the influence of the calibration phantom size was also investigated. The reason behind this was twofold. The first point was that using a calibration phantom of a size similar to the test phantom is too fair for the whole procedure and does not correspond to what would be possible with patients. The second point was that large phantoms are not easy to handle. Therefore, any reduction in the calibration phantom size would ease the calibration procedure. This would be particularly desirable if the procedure has to be repeated frequently. The largest calibration phantom used (XL) had sizes comparable to the NEMA and contrast phantoms. The two other calibration phantoms (L and M) had reduced sizes while the cylindrical shape was maintained.

The accuracy of the activity measurement is a very important parameter in this part of our study. As the various departments were not equipped to measure aliquots, the local radionuclide calibrator was used. It is important to note that the activities of the NEMA, contrast phantom, and S phantom were likewise measured. Therefore, the overall reproducibility of the radionuclide calibrator was of much more concern than its accuracy. The daily quality control procedure of the radionuclide calibrator was expected to reduce the error resulting from fluctuations in time to below 3%. Moreover, the same operator always performed all the measurements.

Due to the presence of the artifacts, ROIs of various diameters were drawn on the calibration phantoms. When the ROI diameter equalled the physical diameter of the phantom, the reconstructed activity in contrast, NEMA, and S phantoms was systematically the highest (Figure 3). OSEM is a conservative process in terms of the number of total reconstructed counts. Therefore, the edge overshoot would result in an underestimation of the body part, and the sensitivity would be found lower if the overshoot is not included in the ROI drawn on the calibration phantom. Fluctuations of the reconstructed activity with ROI size were observed for all calibration phantoms (Figure 3). They can easily be related to the oscillation artifacts. The amplitudes of these oscillation intensities increased with the decrease in phantom size, as did the fluctuations of the reconstructed activity (Figure 3).

The reconstructed activities depended clearly on the calibration phantom with differences between the phantoms starting at a low level (but within expected measurement errors), 2% to 3% for Brightview, and increasing to as much as 15% to 20% for Infinia. The calibration phantom resulting in the lowest error differed between the systems and depended on the test phantom considered. The reconstructed activity was higher by 0% to 5% in the contrast phantom than in the NEMA phantom. This difference lies within the experimental errors. Therefore, Teflon seemed not to preclude quantification in the NEMA phantom.

In the S phantom, the reconstructed activity was systematically underestimated, although over- and underestimations were observed for the contrast and NEMA phantoms. The use of a 1% threshold for the drawing of the ROI should have ensured that all counts are included in the ROI [21]. We have no definitive explanation for the underestimation of the S phantom activity.

Considering the results with the contrast and NEMA phantoms, quantification within 10% or even 5% error seems to be feasible, and further refinement of the calibration parameters would eventually improve the accuracy. Previous studies using different systems, isotopes, and phantoms obtained accuracies in the range 0% to 20% ([21] and references therein, [22]). With patients, Willowson et al. [22] obtained an average error of 1%, a per-patient error of less than 5% in 11 out of 12 patients, and an error of 7.4% in the 12th patient. These studies used older cameras that, with the exception of Infinia, are no longer on the market; some used separated stand-alone SPECT and CT systems, and the data were reconstructed with a locally developed software. With Symbia T series and Flash3D, an overall quantification error better than 7% in phantoms and around 1% in patients was reported [4]. Some per-patient errors were as high as 17%, but the per-patient error was below 10% for 13 out of 16 patients. Finally, it is interesting to remember that Hughes et al. [24] concluded that ‘no significant differences were observed between image resolutions when data acquired from different cameras were reconstructed with an independent algorithm. However, different manufacturers’ reconstruction algorithms produced myocardial wall thickness that differed by up to about 110%.’ In a very recent study [25], the same authors concluded that there were no differences in the figures of merit parameters when data recorded with different SPECT-CT systems were reconstructed with their own software but that significant differences existed when the manufacturers’ reconstruction software was used.

### Reproducibility

This study used several imaging systems located in different departments. Under these conditions, it is very difficult to evaluate the experimental error by repeating the measurements several times. The same operators performed all experiments. Nevertheless, the overall reproducibility needs to be assessed in some way. To this end, it was decided to repeat some experiments twice with a short delay, with a longer delay, after changing one parameter, or on a second camera of the same model.

Two successive SPECT acquisitions of the contrast phantom were performed with Symbia T6. Also, the contrast phantom acquisition was repeated with this Symbia T6 twice with a 1-month interval. One of these acquisitions included 256 projections instead of 128, and therefore, the total number of counts was almost double. In all cases, the contrast recoveries differed by less than 10% for rods larger than 10 mm and by less than 20% for most of the smallest rods. With the Siemens T6 system, a shorter acquisition of the NEMA phantom resulted in four times fewer acquired counts, but the values for the residues in the inserts differed from those obtained with the high count acquisition by less than 0.5%. All these repeated experiments indicated that the acquisition parameters, and particularly the number of acquired counts, ensured good short- and long-term reproducibility. Therefore, the results reported in this study are likely to represent effective differences in performance between the four investigated systems.

Imaging the NEMA phantom with a second Brightview camera or a Symbia T2 also led to very reproducible results. Differences in residual fractions were less than 1% between the two Brightview systems and less than 2.5% between Symbia T6 and T2. Also, the attenuation coefficients obtained with the two Brightview or the two Symbia systems were identical in water and differed by less than 3% in Teflon. In the preliminary study (Additional file 2), it was observed that contrast recoveries obtained at a 4-year interval with two cameras of the same model differed by less than 10% for all but one of the hot rods and for the largest cold rods. Therefore, the data issued from the use of a second camera of the same model tended to demonstrate that the results were not particular to the specific camera used for this study.

The CF determination is a crucial step in the quantification procedure. The repeatability and reproducibility of this step were assessed with Symbia T6 and the M phantom. This choice resulted from easy access to this camera, the fact that decay correction was not performed in the Flash3D reconstruction software but in a separate procedure, and the highest intensity of the artifacts for the M phantom. The likelihood of the highest variability was therefore expected when considering the M phantom and Symbia T6. The repeatability was found to be better than 0.5%. The differences between CFs obtained at short interval were around 3.4%. Such small differences are similar to the reproducibility of the radionuclide calibrators. After 10 months, the differences were 5.0% to 6.6% for all ROIs except the 60% ROI, for which the value was as high as 13.6%. However, the limits of this ROI corresponded to a region of a rapid variation in the reconstructed counts resulting from the oscillation artifacts (Figures 4 and 5). This observation stresses again the need for future work devoted to suppression of these artifacts for more accurate quantification in SPECT-CT.

## Conclusions

The four SPECT-CT systems and their 3D iterative reconstruction with attenuation and scatter corrections and resolution recovery achieved satisfactorily good attenuation and scatter correction and improved contrast for small structures. In objects whose dimensions exceed the SPECT spatial resolution by several times, quantification based on a calibration procedure similar to the one used in PET seems to be feasible within 10% error limits and even below if a fine-tuning of all acquisition and reconstruction parameters is performed. A partial volume effect correction strategy remains necessary for the smaller structures. Reconstruction artifacts were observed for all systems. They are a clear handicap on the road towards accurate quantification in SPECT and should be the focus of further studies in reconstruction tomography.

## Abbreviations

CF: conversion factor; CRC: contrast recovery coefficient; CT: computerized tomography; ESSE: effective source scatter estimation; FBP: filtered back-projection; FWHM: full width at half maximum; HU: Hounsfield unit; L: cylindrical phantom of 8-cm height and 9.4-cm diameter; M: cylindrical phantom of 8-cm height and 5.4-cm diameter; MAPEM: maximum a posteriori expectation maximization; MLEM: maximum likelihood expectation maximization; MLEMit: equivalent number of MLEM iterations; NEMA: National Electrical Manufacturers Association; OSEM: ordered subsets expectation maximization; PET: positron emission tomography; ROI: region of interestx; S: cylindrical phantom of 8-cm height and 1.6-cm diameter; SPECT: single-photon emission tomography; TEW: triple energy window; XL: cylindrical phantom of 30-cm height and 20-cm diameter.

## Competing interests

The authors declare that they have no competing interests.

## Authors’ contributions

AS conceived of the study, participated in its design and coordination and in the measurements, and drafted the manuscript. DN carried out all measurements and data processing, and helped draft the manuscript. CB participated in the study design and coordination and in the measurements, and helped draft the manuscript. All authors read and approved the final manuscript.

## Supplementary Material

Additional file 1 **Figure S1.** Residual fraction in cold inserts of the NEMA NU 2–1994 phantom without scatter correction. Results for the three non-emitting air (square), water (circle), and Teflon (triangle) inserts as a function of the number of iterations with eight subsets for the four SPECT-CT systems. (A) Philips Brightview XCT. (B) General Electric Discovery NM/CT 670. (C) General Electric Infinia Hawkeye 4. (D) Siemens Symbia T6. All were reconstructions with attenuation correction and resolution recovery. Click here for file

Additional file 2 **Preliminary study.** A preliminary study using FBP and Chang attenuation correction to reconstruct the data obtained in the present study. Click here for file

Additional file 3 **Figure S6.** Contrast recovery in function of the number of iterations for the Philips Brightview XCT. Reconstructions were performed with Philips Astonish including attenuation and scatter correction and resolution recovery and eight subsets. (A, B) Hot rods. (C, D) Cold rods. (A, C) Full ROI. (B ,D) Half ROI. Click here for file

Additional file 4 **Figure S7.** Contrast recovery in function of the number of iterations for the General Electric Discovery NM/CT670. Reconstructions were performed with General Electric Evolution for Bone including attenuation and scatter correction and resolution recovery and eight subsets. (A, B) Hot rods. (C, D) Cold rods. (A, C) Full ROI. (B ,D) Half ROI. Click here for file

Additional file 5 **Figure S8.** Contrast recovery in function of the number of iterations for the General Electric Infinia Hawkeye 4. Reconstructions were performed with General Electric Evolution for Bone including attenuation and scatter correction and resolution recovery and eight subsets. (A, B) Hot rods. (C, D) Cold rods. (A, C) Full ROI. (B ,D) Half ROI. Click here for file

Additional file 6 **Figure S9.** Contrast recovery as a function of the number of iterations for the Siemens Symbia T6. Reconstructions were performed with Siemens. Flash3D including attenuation and scatter correction and resolution recovery and eight subsets. (A, B) Hot rods. (C, D) Cold rods. (A, C) Full ROI. (B ,D) Half ROI. Click here for file

Additional file 7 **Figure S10.** Contrast recovery with and without post filter for the Siemens Symbia T6. Reconstructions were performed with Siemens Flash3D including attenuation and scatter correction and resolution recovery and eight subsets. (A, B) Hot rods. (C, D) Cold rods. (A, C) Full ROI. (B ,D) Half ROI. Click here for file

Additional file 8 **Figure S11.** Transverse and coronal slices of the L phantom obtained after 24 iterations. (A, E) Philips Brightview XCT and Astonish. (B, F) General Electric Discovery NM/CT 670 and Evolution for Bone. (C, G) General Electric Infinia Hawkeye-4 and Evolution for Bone. (D, H) Siemens Symbia T6 and Flash3D. All were reconstructions with eight subsets. Hot iron color scale from 0 to 110% of slice maximum. Click here for file

Additional file 9 **Figure S12.** SPECT and CT fused slices of the contrast phantom imaged with the Siemens Symbia T6. SPECT reconstructions were performed with Siemens Flash3D including attenuation and scatter correction and resolution recovery, 24 iterations and eight subsets. (A) Transverse slice of the hot rods. (B) Transverse slice of the cold rods. (C) Coronal slice. The part at the bottom of the coronal slice is a grid and was not used in this work. Click here for file
