# Supplementary material for: Quantitative capabilities of four state-of-the-art SPECT-CT cameras
Source: EJNMMI Res. 2012 Aug 27;2:45. doi: 10.1186/2191-219X-2-45 (PMC3469367; doi:10.1186/2191-219X-2-45)

**Supplementary Figure 1. Residual fraction in cold inserts of the NEMA NU 2-1994 phantom without scatter correction.** Results for the three non-emitting air (square), water (circle), and Teflon (triangle) inserts as a function of the number of iterations with eight subsets for the four SPECT-CT systems. (A) Philips Brightview XCT. (B) General Electric Discovery NM/CT 670. (C) General Electric Infinia Hawkeye 4. (D) Siemens Symbia T6. All are reconstructions with attenuation correction and resolution recovery.

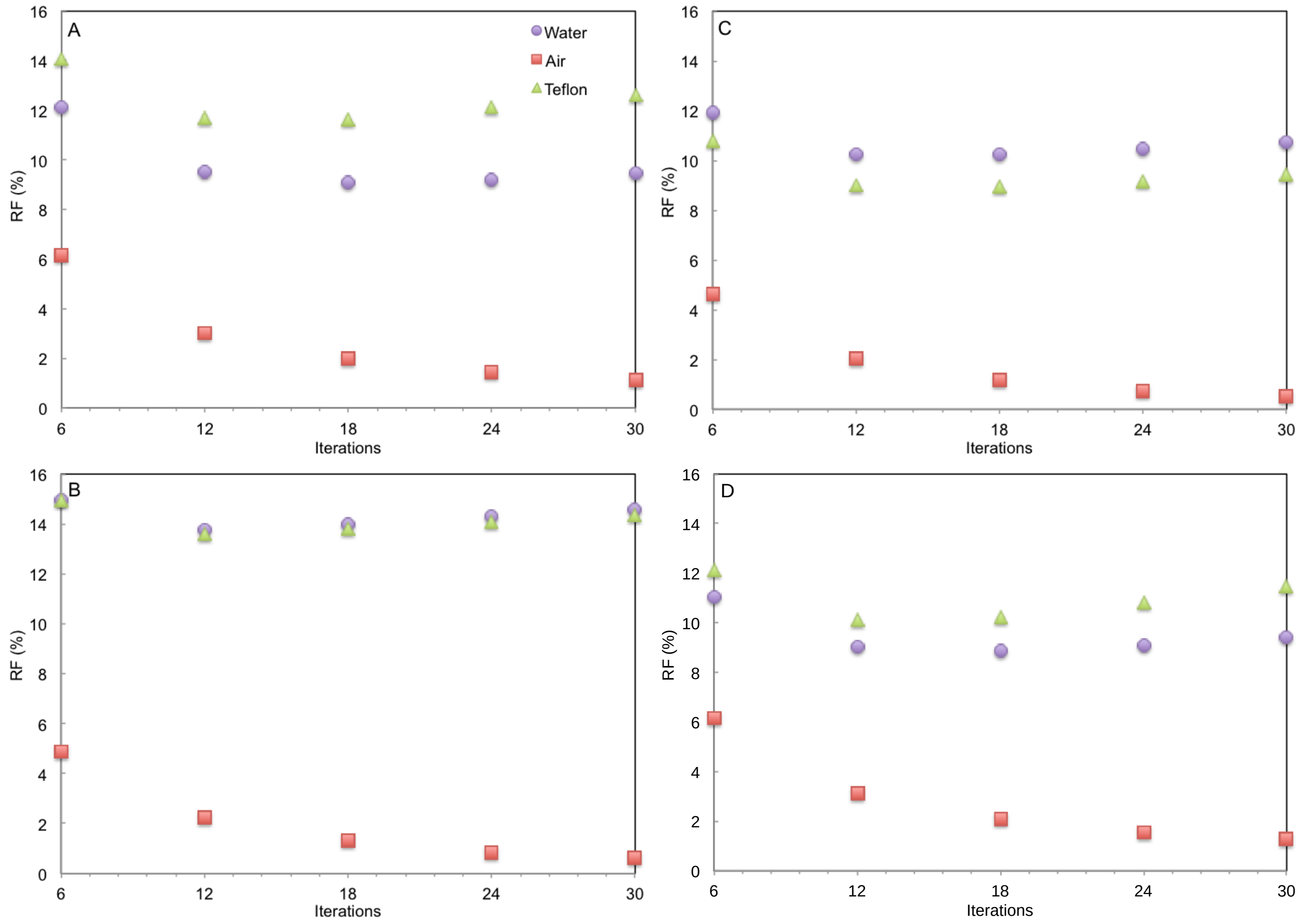

Supplement: Additional file 1 — Figure S1. Residual fraction in cold inserts of the NEMA NU 2–1994 phantom without scatter correction. Results for the three non-emitting air (square), water (circle), and Teflon (triangle) inserts as a function of the number of iterations with eight subsets for the four SPECT-CT systems. (A) Philips Brightview XCT. (B) General Electric Discovery NM/CT 670. (C) General Electric Infinia Hawkeye 4. (D) Siemens Symbia T6. All were reconstructions with attenuation correction and resolution recovery. [file 2191-219X-2-45-S1.pdf]
