# Supplementary material for: Quantitative capabilities of four state-of-the-art SPECT-CT cameras
Source: EJNMMI Res. 2012 Aug 27;2:45. doi: 10.1186/2191-219X-2-45 (PMC3469367; doi:10.1186/2191-219X-2-45)

Preliminary study

Aim

A preliminary study was conducted in order to compare the contrast performance of the four SPECT-CT and older stand-alone SPECT cameras regardless of the iterative reconstruction and the spatial resolution correction. Filtered backprojection is expected not to depend on particular implementation [1] and was therefore used to reconstruct all data.

Methods

The contrast phantom was imaged over the past five years by eight stand-alone dual-head SPECT cameras fitted with their low-energy (LE) high resolution (HR) or ultra-high resolution (UHR) collimators. These were Elscint Helix (UHR), General Electric Magicam (HR), Siemens e-cam (HR) and Multispect-2 (HR), Sopha Medical Vision DST (HR), DST-XL (UHR), and DST-XLi (HR and UHR). The acquisition parameters were identical to those used in the main study. These data were reconstructed with FBP and a ramp filter at Nyquist frequency and Chang attenuation correction, with broad beam attenuation coefficient chosen to make profiles of phantom uniform part flat, was used to correct for attenuation and partly for scatter. The data obtained with the four SPECT-CT cameras were likewise reconstructed. The twelve image sets were analysed as described in the main study and the contrast recovery coefficients (CRC) were computed for each rod and both ROIs (full ROI and half ROI).

To assess the reproducibility, the contrast phantom was scanned consecutively three times with the Symbia T6 and also three times at one-month interval. One of these acquisitions was performed with 256 projections instead of 128 but with the same number of counts in the first projection. For this particular acquisition the total number of counts was almost doubled. Four years earlier, the contrast phantom had been scanned with identical parameters on a stand-alone General Electric Infinia camera. All these data were processed as indicated above.

Results

The mean contrast recovery and the minimum and maximum contrast recovery were determined for the eight data sets of the stand-alone dual-head SPECT cameras. They are plotted as continuous lines in Supplementary Figures 2 to 5 for the hot (Supplementary Figures 2 and 3) and cold (Supplementary Figures 4 and 5) contrasts and full (Supplementary Figures 2 and 4) or half (Supplementary Figures 3 and 5) ROI.
The results obtained with the four SPECT-CT systems of the main study are plotted as individual points in Supplementary Figures 2 to 5. Most of these contrast recovery values were situated between the mean and maximum lines with many values closer to the mean line than to the maximum line. The other values were below the mean line but no value was below the minimal line.

For the three consecutive acquisitions, the contrast recovery differed by less than 10% for rods larger than 10 mm. For the smaller rods, the difference was larger but still below 20% with the exception of one cold rod. When the scan interval was a one month, the differences in contrast were less than to 10% for inserts larger than 8 mm. For the smaller hot (cold) rods, the difference increased and amounted to 20 (60)% for the smallest ones and the half ROI. Contrast recoveries for data obtained four years earlier with a stand-alone Infinia camera differed from those obtained with the Infinia Hawkeye used for the main study by less than 10% for all but one of the hot rods and for the largest cold rods.

Discussion

Before attributing eventual differences in contrast recovery to differences of reconstruction algorithm we felt that it was important to compare the contrast performance of the four systems without their full 3D iterative reconstruction with resolution recovery. The data of the contrast phantom were therefore reconstructed using FBP and Chang attenuation correction. Data of the same phantom recorded over the past five years on eight different dual-head stand-alone SPECT cameras with the same methodology were likewise reconstructed. The results (Supplementary Figures 2 to 5) showed very similar hot and cold contrast recovery for these four state-of-the-art SPECT systems and no definitive improvements over their predecessors. They also demonstrated that when two cameras of the same model were tested the contrasts were very similar, with the exception of the smallest cold rods. However, this is more likely to result from noise than from performance difference.

When repeating the whole process (acquisition and processing) consecutively, with a one-month interval or with a double number of acquired counts, the contrast recoveries differed by less than 10% for rods larger than 10 mm and by less than 20% for most of the smallest rods. These repeated experiments indicated that the acquisition parameters, and in particular the number of acquired counts, ensured good short- and long-term reproducibility. Therefore, the results reported in this preliminary study are likely to represent effective performance of the investigated cameras.

Reference

1. Seret A, Forthomme J: **Comparison of Different Types of Commercial Filtered Backprojection and Ordered-Subset Expectation Maximization SPECT Reconstruction Software.** *J Nucl Med Technol* 2009, 39:179–187.

Supplementary Figure 2: Contrast recovery with the full ROI for the hot rods of the contrast phantom. Data were reconstructed with FBP and Chang attenuation correction. The continuous lines represent the mean, minimum, and maximum contrast recovery as observed for eight different dual-head stand-alone SPECT cameras. Individual points are the values obtained with the SPECT-CT systems under investigation. (A) Philips Brightview XCT. (B) General Electric Discovery NM/CT 670. (C) General Electric Infinia Hawkeye 4. (D) Siemens Symbia T series. The eight stand-alone cameras were the following: Elscint Helix, General Electric Magicam, Siemens e-cam and Multispect-2, Sopha Medical Vision DST, DST-XL, DST-Xli (two cameras).

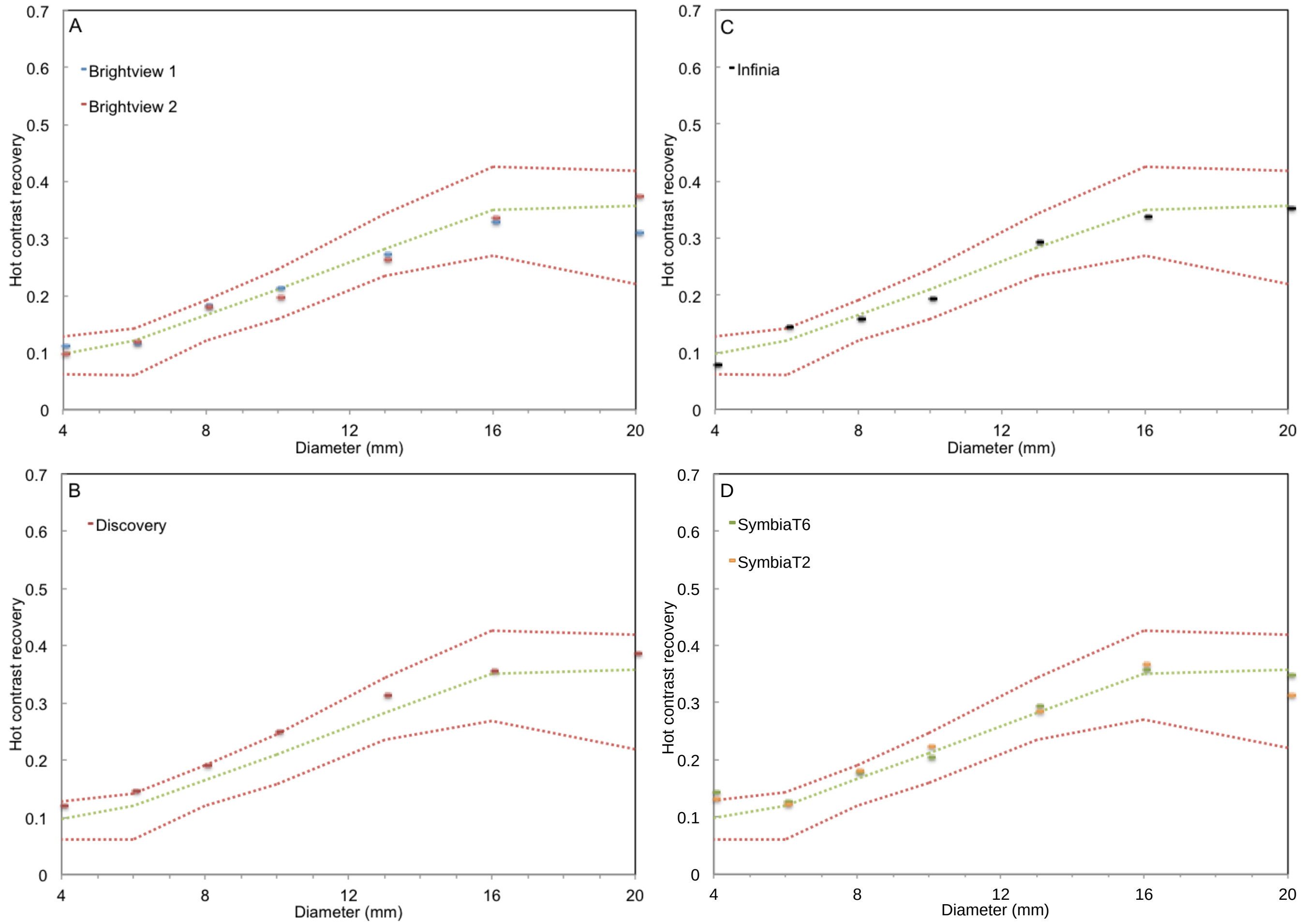

Supplementary Figure 3: Contrast recovery with the half ROI for the hot rods of the contrast phantom. Data were reconstructed with FBP and Chang attenuation correction. The continuous lines represent the mean, minimum and maximum contrast recovery as observed for eight different dual-head stand-alone SPECT cameras. Individual points are the values obtained with the SPECT-CT systems under investigation. (A) Philips Brightview XCT. (B) General Electric Discovery NM/CT 670. (C) General Electric Infinia Hawkeye 4. (D) Siemens Symbia T series. The eight stand-alone cameras were the following: Elscint Helix, General Electric Magicam, Siemens e-cam and Multispect-2, Sopha Medical Vision DST, DST-XL, DST-Xli (two cameras).

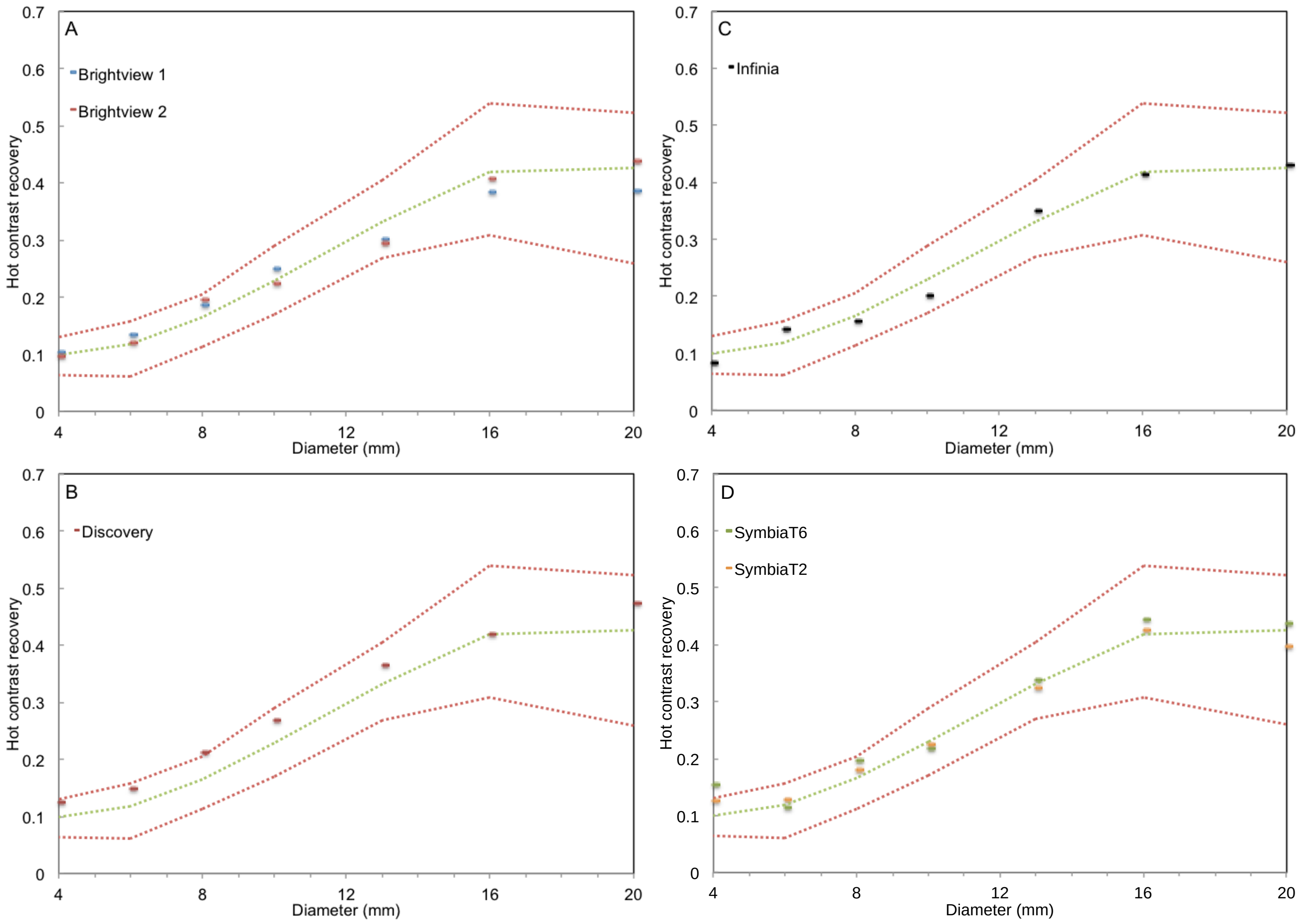

Supplementary Figure 4: Contrast recovery with the full ROI for the cold rods of the contrast phantom. Data were reconstructed with FBP and Chang attenuation correction. The continuous lines represent the mean, minimum, and maximum contrast recovery as observed for eight different dual-head stand-alone SPECT cameras. Individual points are the values obtained with the SPECT-CT systems under investigation. (A) Philips Brightview XCT. (B) General Electric Discovery NM/CT 670. (C) General Electric Infinia Hawkeye 4. (D) Siemens Symbia T series. The eight stand-alone cameras were the following: Elscint Helix, General Electric Magicam, Siemens e-cam and Multispect-2, Sopha Medical Vision DST, DST-XL, DST-Xli (two cameras).

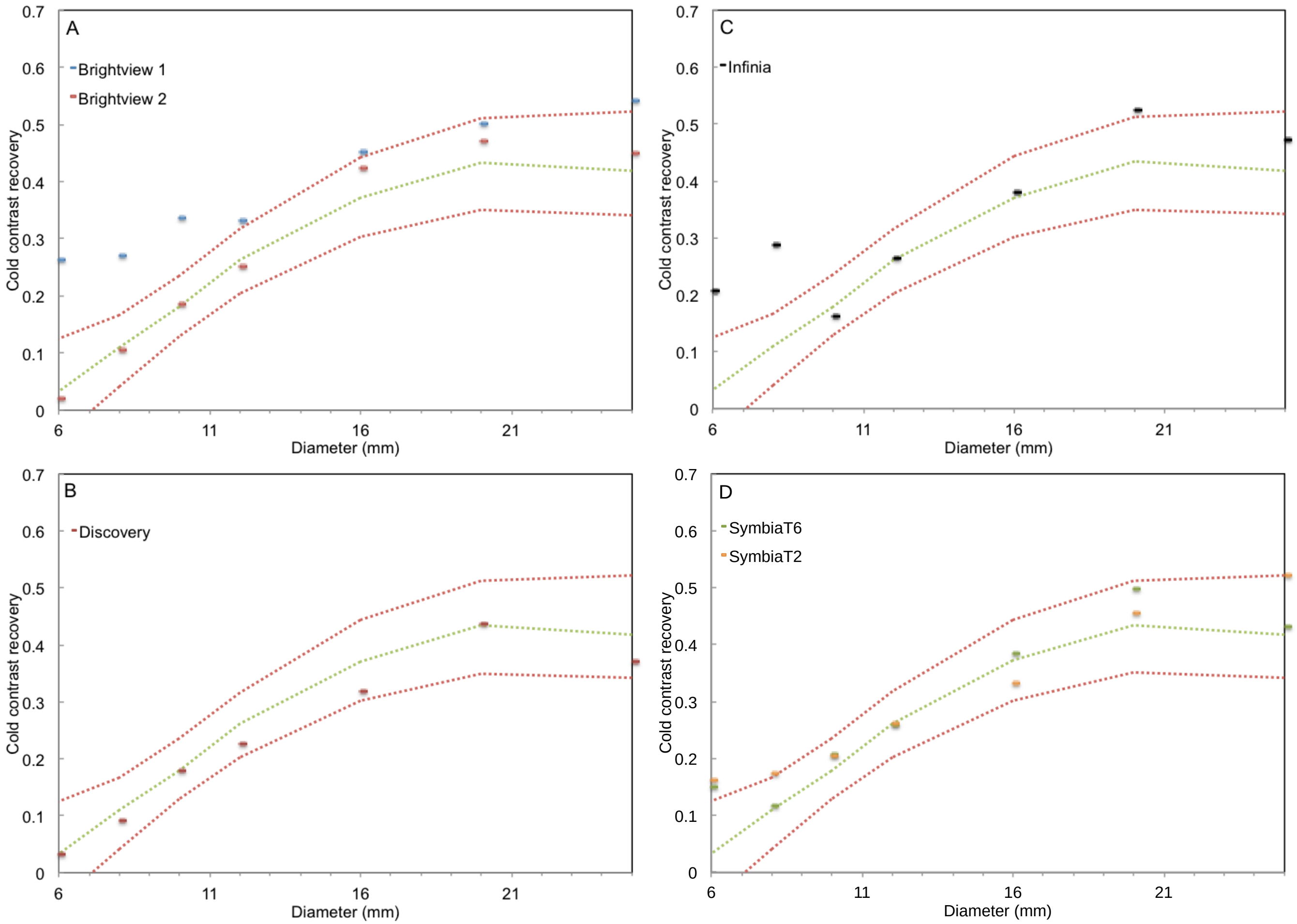

Supplementary Figure 5: Contrast recovery with the half ROI for the cold rods of the contrast phantom. Data were reconstructed with FBP and Chang attenuation correction. The continuous lines represent the mean, minimum, and maximum contrast recovery as observed for eight different dual-head stand-alone SPECT cameras. Individual points are the values obtained with the SPECT-CT systems under investigation. (A) Philips Brightview XCT. (B) General Electric Discovery NM/CT 670. (C) General Electric Infinia Hawkeye 4. (D) Siemens Symbia T series. The eight stand-alone cameras were the following: Elscint Helix, General Electric Magicam, Siemens e-cam and Multispect-2, Sopha Medical Vision DST, DST-XL, DST-Xli (two cameras).

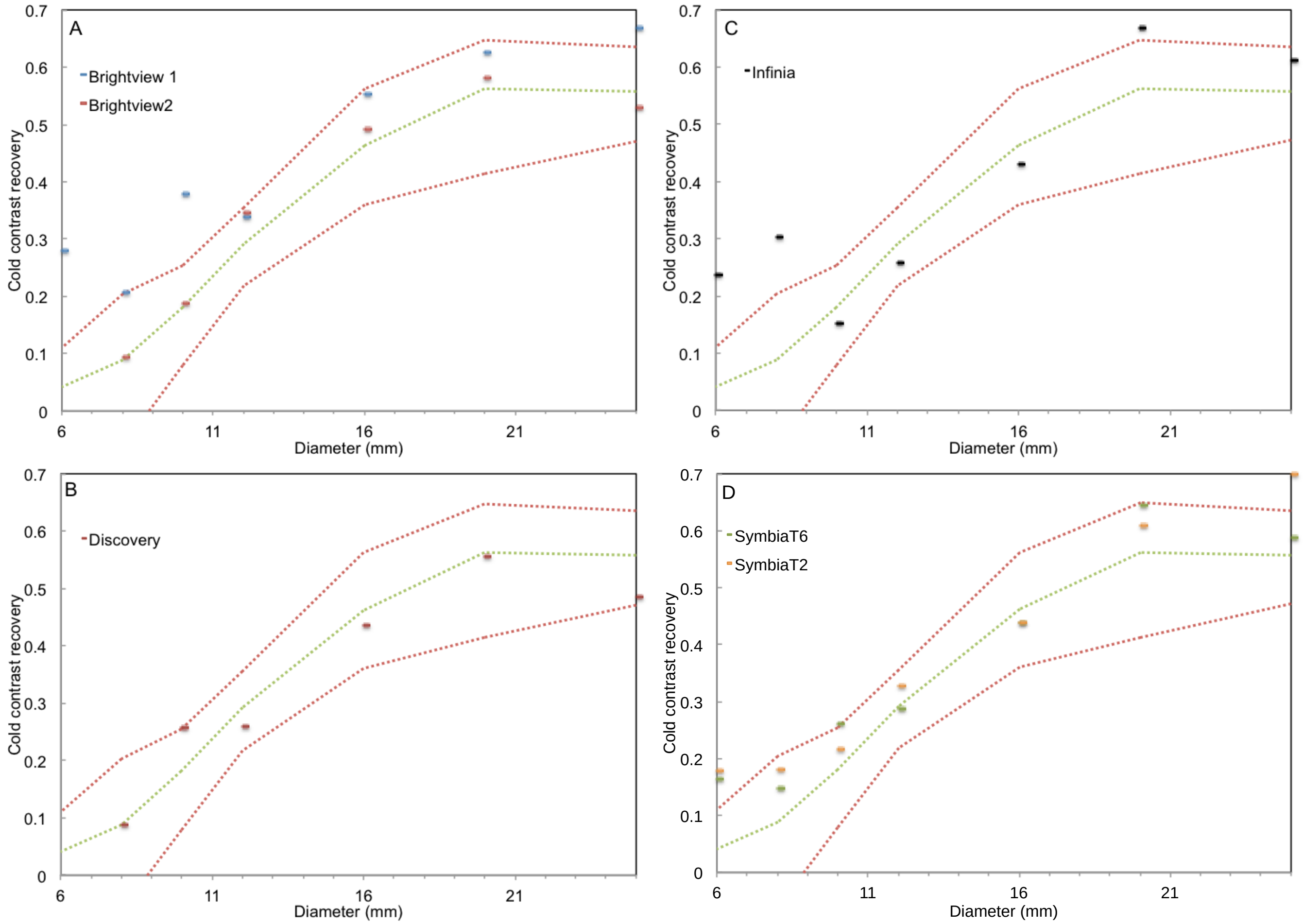

Supplement: Additional file 2 — Preliminary study. A preliminary study using FBP and Chang attenuation correction to reconstruct the data obtained in the present study. [file 2191-219X-2-45-S2.pdf]
