# Supplementary material for: Quantitative capabilities of four state-of-the-art SPECT-CT cameras
Source: EJNMMI Res. 2012 Aug 27;2:45. doi: 10.1186/2191-219X-2-45 (PMC3469367; doi:10.1186/2191-219X-2-45)

Supplementary Figure 6. Contrast recovery in function of the number of iterations for Philips Brightview XCT. Reconstructions were performed with Philips Astonish including attenuation and scatter correction and resolution recovery and eight subsets. (A, B) Hot rods. (C, D) Cold rods. (A, C) Full ROI. (B, D) Half ROI.

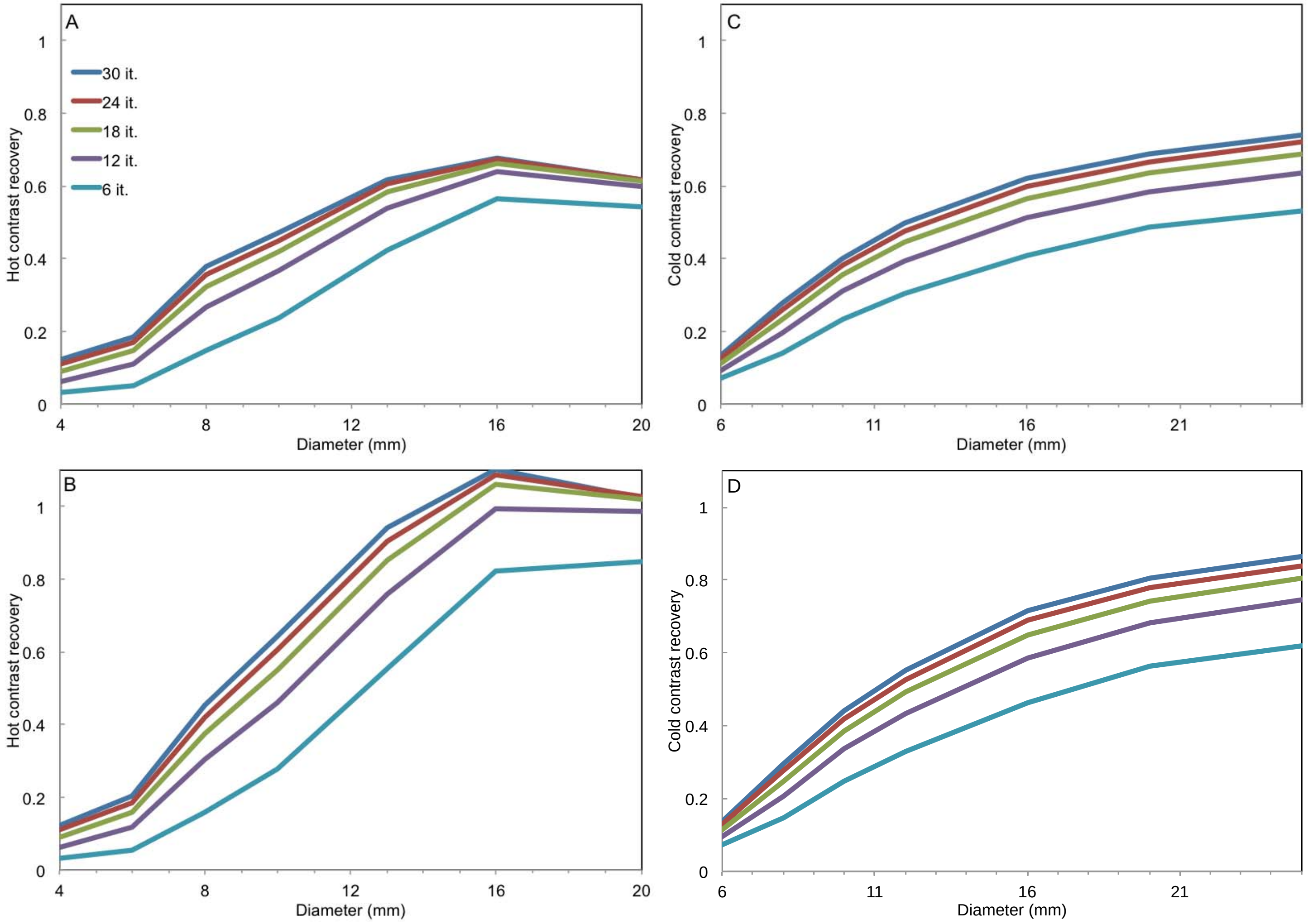

Supplement: Additional file 3 — Figure S6. Contrast recovery in function of the number of iterations for the Philips Brightview XCT. Reconstructions were performed with Philips Astonish including attenuation and scatter correction and resolution recovery and eight subsets. (A, B) Hot rods. (C, D) Cold rods. (A, C) Full ROI. (B ,D) Half ROI. [file 2191-219X-2-45-S3.pdf]
