# Supplementary material for: Quantitative capabilities of four state-of-the-art SPECT-CT cameras
Source: EJNMMI Res. 2012 Aug 27;2:45. doi: 10.1186/2191-219X-2-45 (PMC3469367; doi:10.1186/2191-219X-2-45)

Supplementary Figure 10. Contrast recovery with and without post filter for Siemens Symbia T6. Reconstructions were performed with Siemens Flash3D including attenuation and scatter correction and resolution recovery and eight subsets. (A, B) Hot rods. (C, D) Cold rods. (A, C) Full ROI. (B, D) Half ROI.

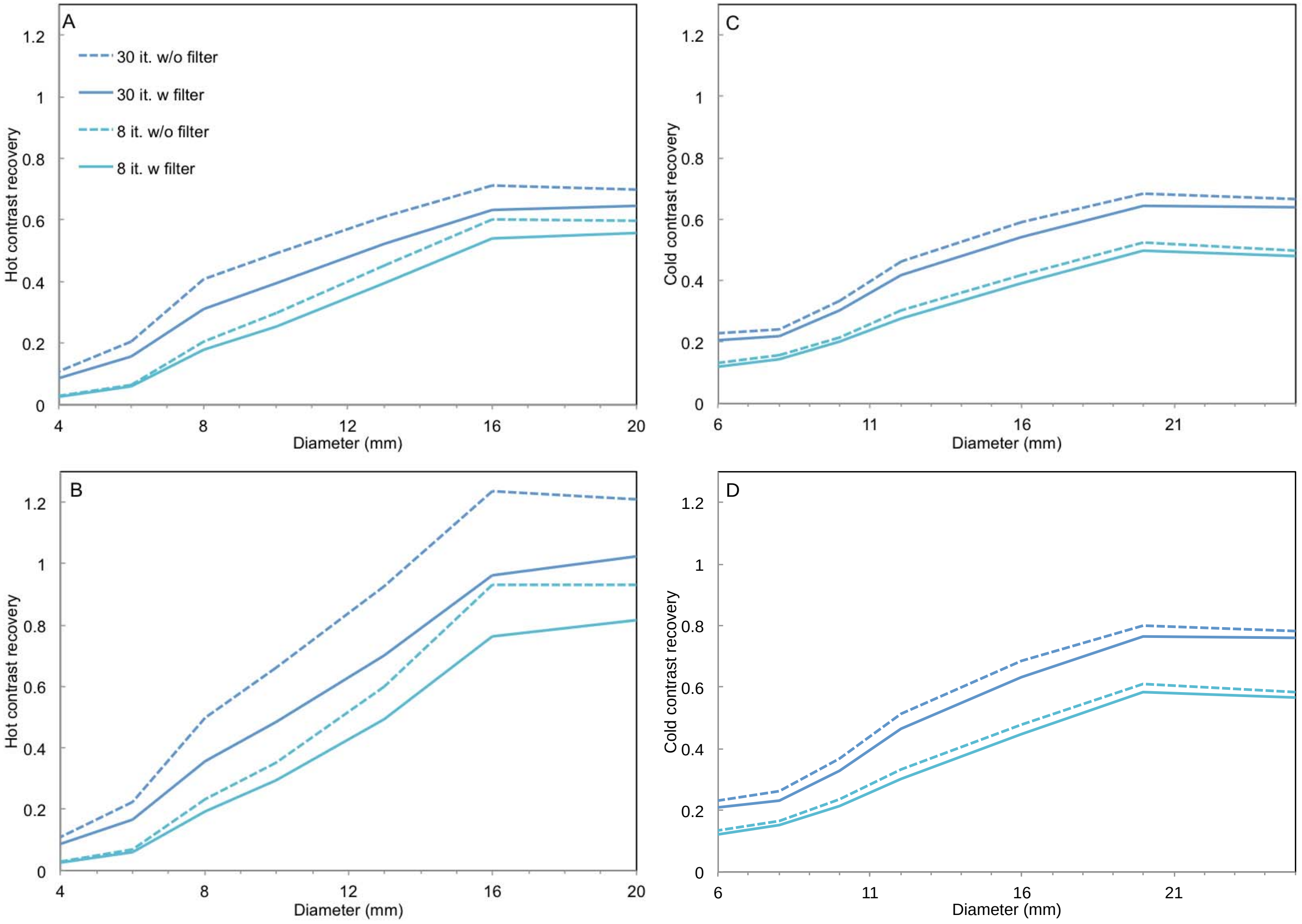

Supplement: Additional file 7 — Figure S10. Contrast recovery with and without post filter for the Siemens Symbia T6. Reconstructions were performed with Siemens Flash3D including attenuation and scatter correction and resolution recovery and eight subsets. (A, B) Hot rods. (C, D) Cold rods. (A, C) Full ROI. (B ,D) Half ROI. [file 2191-219X-2-45-S7.pdf]
