# Supplementary material for: Quantitative capabilities of four state-of-the-art SPECT-CT cameras
Source: EJNMMI Res. 2012 Aug 27;2:45. doi: 10.1186/2191-219X-2-45 (PMC3469367; doi:10.1186/2191-219X-2-45)

Supplementary Figure 11. Transverse and coronal slices of the L phantom obtained after 24 iterations. (A, E) Philips Brigthview XCT and Astonish. (B, F) General Electric Discovery NM/CT 670 and Evolution for Bone. (C, G) General Electric Infinia Hawkeye-4 and Evolution for Bone. (D, H) Siemens Symbia T6 and Flash3D. All were reconstructions with eight subsets. Hot iron color scale from 0 to 110% of slice maximum.

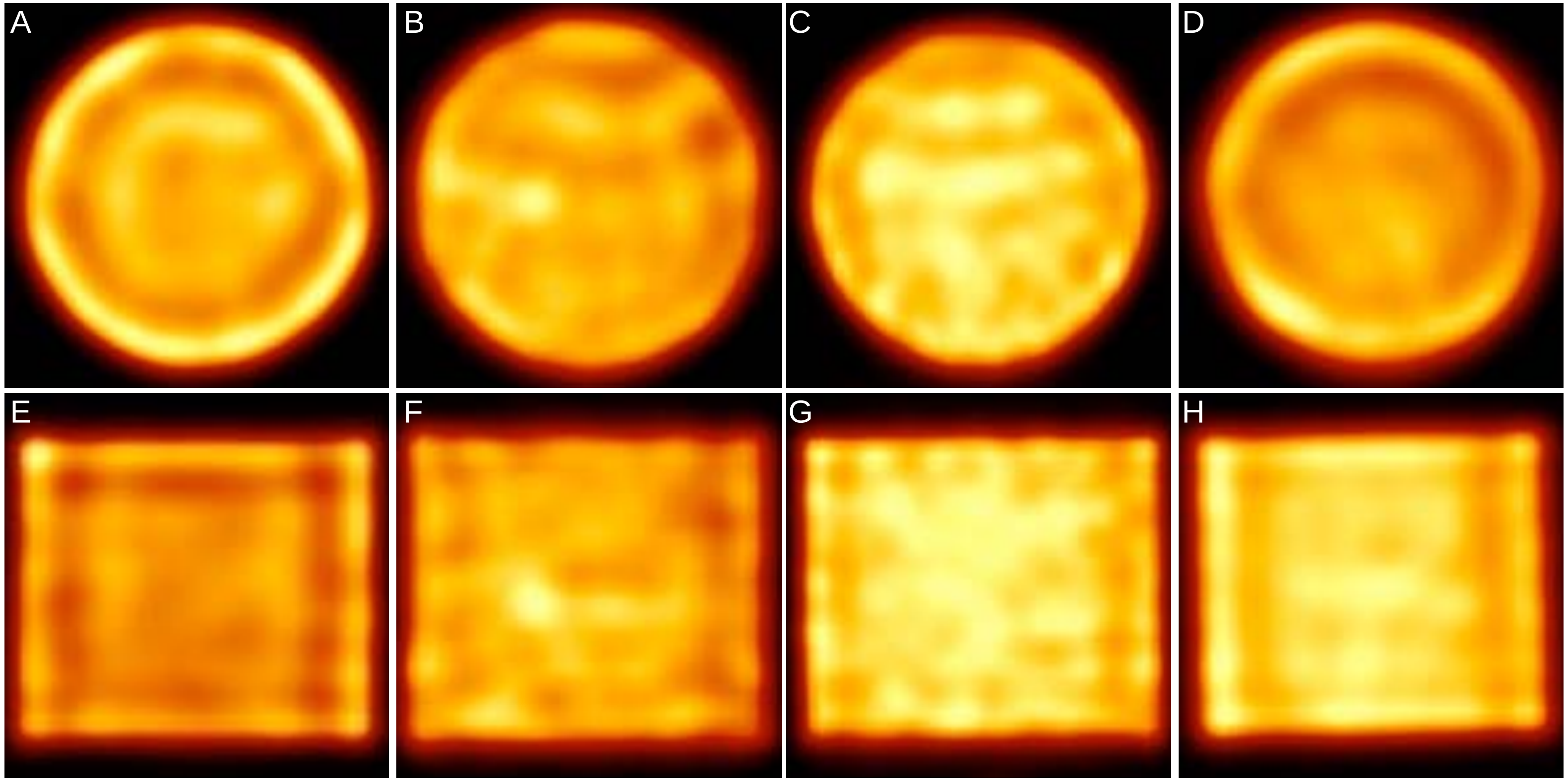

Supplement: Additional file 8 — Figure S11. Transverse and coronal slices of the L phantom obtained after 24 iterations. (A, E) Philips Brightview XCT and Astonish. (B, F) General Electric Discovery NM/CT 670 and Evolution for Bone. (C, G) General Electric Infinia Hawkeye-4 and Evolution for Bone. (D, H) Siemens Symbia T6 and Flash3D. All were reconstructions with eight subsets. Hot iron color scale from 0 to 110% of slice maximum. [file 2191-219X-2-45-S8.pdf]
