# Supplementary material for: Quantitative capabilities of four state-of-the-art SPECT-CT cameras
Source: EJNMMI Res. 2012 Aug 27;2:45. doi: 10.1186/2191-219X-2-45 (PMC3469367; doi:10.1186/2191-219X-2-45)

Supplementary Figure 12. SPECT and CT fused slices of the contrast phantom imaged with Siemens Symbia T6. SPECT reconstructions were performed with Siemens Flash3D including attenuation and scatter correction and resolution recovery, 24 iterations and eight subsets. (A) Transverse slice of the hot rods. (B) Transverse slice of the cold rods. (C) Coronal slice. The part at the bottom of the coronal slice is a grid and was not used in this work.

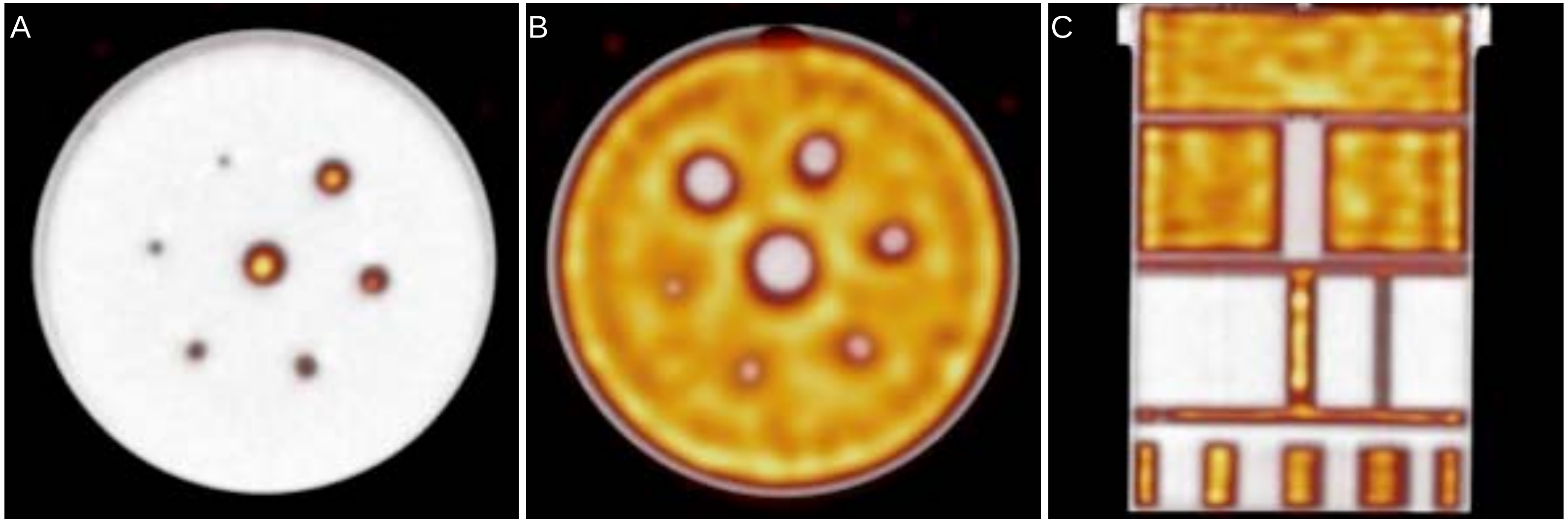

Supplement: Additional file 9 — Figure S12. SPECT and CT fused slices of the contrast phantom imaged with the Siemens Symbia T6. SPECT reconstructions were performed with Siemens Flash3D including attenuation and scatter correction and resolution recovery, 24 iterations and eight subsets. (A) Transverse slice of the hot rods. (B) Transverse slice of the cold rods. (C) Coronal slice. The part at the bottom of the coronal slice is a grid and was not used in this work. [file 2191-219X-2-45-S9.pdf]
